# The Influence of Soy Isoflavones and Soy Isoflavones with Inulin on Kidney Morphology, Fatty Acids, and Associated Parameters in Rats with and without Induced Diabetes Type 2

**DOI:** 10.3390/ijms25105418

**Published:** 2024-05-16

**Authors:** Kamila Misiakiewicz-Has, Dominika Maciejewska-Markiewicz, Dagmara Szypulska-Koziarska, Agnieszka Kolasa, Barbara Wiszniewska

**Affiliations:** 1 Department of Histology and Embryology, Pomeranian Medical University, Powstańców Wielkopolskich 72, 70-111 Szczecin, Poland; dagmara.szypulska.koziarska@pum.edu.pl (D.S.-K.); agnieszka.kolasa@pum.edu.pl (A.K.); barbara.wiszniewska@pum.edu.pl (B.W.); 2 Department of Human Nutrition and Metabolomics, Pomeranian Medical University in Szczecin, 70-204 Szczecin, Poland; dominika.maciejewska.markiewicz@pum.edu.pl

**Keywords:** diabetes, soy isoflavones, inulin, kidney, fatty acids, morphology

## Abstract

Diabetes mellitus resulting from hyperglycemia stands as the primary cause of diabetic kidney disease. Emerging evidence suggests that plasma concentrations of soy isoflavones, substances with well-established antidiabetic properties, rise following supplemental inulin administration. The investigation encompassed 36 male Sprague–Dawley (SD) rats segregated into two cohorts: non-diabetic and diabetic, induced with type 2 diabetes (high-fat diet + two intraperitoneal streptozotocin injections). Each cohort was further divided into three subgroups (n = 6): control, isoflavone-treated, and isoflavone plus inulin-treated rats. Tail blood glucose and ketone levels were gauged. Upon termination, blood samples were drawn directly from the heart for urea, creatinine, and HbA1c/HbF analyses. One kidney per rat underwent histological (H-E) and immunohistochemical assessments (anti-AQP1, anti-AQP2, anti-AVPR2, anti-SLC22A2, anti-ACC-alpha, anti-SREBP-1). The remaining kidney underwent fatty acid methyl ester analysis. Results unveiled notable alterations in water intake, body and kidney mass, kidney morphology, fatty acids, AQP2, AVPR2, AcetylCoA, SREBP-1, blood urea, creatinine, and glucose levels in control rats with induced type 2 diabetes. Isoflavone supplementation exhibited favorable effects on plasma urea, plasma urea/creatinine ratio, glycemia, water intake, and kidney mass, morphology, and function in type 2 diabetic rats. Additional inulin supplementation frequently modulated the action of soy isoflavones.

## 1. Introduction

One of the defining characteristics of diabetes mellitus is lipid dysmetabolism, which is manifested by dyslipidemia and the accumulation of lipids in various tissues and organs, including the kidney. The pathogenesis of diabetic kidney disease (DKD) involves a complex interplay of factors, including impaired cholesterol metabolism, increased lipid synthesis or uptake, increased oxidation of fatty acids, accumulation of lipid droplets, and an imbalance in biologically active sphingolipids [1]. Diabetes mellitus due to hyperglycemia is the leading cause of DKD and end-stage renal disease (ESRD) and an increased risk of cardiovascular mortality in many parts of the world [2]. The number of people with diabetes, 90–95% of whom have type 2 diabetes, could reach 35 million by 2035, and 40% of these patients will develop chronic kidney disease, a precursor to DKD [3].

Diabetic kidney disease is characterized by both structural and functional abnormalities. The clinical and pathological hallmarks of DKD include a decrease in glomerular filtration rate (GFR), albuminuria, changes in renal morphology (accumulation of extracellular matrix, thickening of basement membranes, hypertrophy and podocyte loss within the glomeruli, arterial hyalinosis), comorbidity of hypertension, and abnormal renal function represented by abnormalities in serum creatinine and serum urea [4]. Serum urea and creatinine are elevated with hyperglycemia in uncontrolled diabetes and often correlate with the severity of kidney damage [5]. Glomerular damage is often associated with hyperfiltration, which is associated with renal hypertrophy and polyuria, followed by increased albuminuria and decreased GFR [6]. Type 2 diabetes is associated with high plasma cholesterol and triglyceride levels and general dyslipidemia, which are independent risk factors for cardiorenal disease progression in humans [7].

As mentioned above, DKD is considered to be a consequence of the hyperglycemia present in diabetes mellitus. However, the accumulation of lipids in podocytes, which line the urinary surface of the capillary tuft in the glomerulus, has also been reported to be associated with the development of DKD [8]. Since the kidney is a target organ for the harmful effects of lipids in diabetes, chronic kidney disease is a form of fatty kidney disease similar to liver disease [1].

It is normal for all cells that lipid metabolism involves the synthesis and breakdown of lipids according to energy needs. Some lipids are oxidized, while others are synthesized and stored. Triacylglycerols are broken down into free fatty acids (FFA), which undergo fatty acid oxidation (FAO). Fatty acid oxidation is the aerobic process of fatty acid breakdown that occurs in mitochondria to obtain energy from fats. During FAO, acetyl coenzyme A (acetyl CoA) is produced along with ATP and water. Genes involved in FAO are downregulated in the kidneys of mice and humans with kidney disease. This is also associated with increased fatty acid synthesis and increased deposition of intracellular lipids [1]. 

The kidney secretes many different small organic electrolytes, which are generally divided into two main classes: organic anions (OAs) and organic cations (OCs). Transepithelial clearance of small cationic molecules in the kidney is mediated by transporters located in the basolateral membrane of proximal tubular cells. In the rat kidney, organic cation transporters—OCT-1 and OCT-2—are responsible for the first step in the active secretion of cations [9]. Organic cation transporters are required for the renal clearance of many exogenous substrates such as toxins or xenobiotics, as well as endogenous compounds such as creatinine, choline, cortisol, prostaglandins, monoamine neurotransmitters or N-methyl-nicotinamide (NMD), or drugs (e.g., metformin) [10]. Proximal tubule function is impaired with disruption of tubular acidification and lysosomal degradation in diabetic nephropathy [10]. 

Dietary phytochemicals can affect the function of many organs involved in the pathogenesis of diabetes (pancreas, liver, muscle, and adipose tissue), as well as organs critical for maintaining glucose homeostasis (gastrointestinal tract, brain, kidney), and may be potent therapeutic agents [11]. Numerous studies have shown that dietary soy isoflavones (such as genistein and daidzein) have beneficial effects on cholesterol levels, body mass index, or adipose tissue volume, and also act as antidiabetic agents [12,13]. The anti-diabetic effect of soy isoflavones is associated with protection of pancreatic beta cells (by increasing beta-cell proliferation and reducing apoptosis), which has been confirmed in animal models of diabetes [14,15]. In addition, soy isoflavones do not accumulate in tissues and have antioxidant properties [16].

There is evidence to suggest that plasma soy isoflavone concentrations increase with additional inulin supplementation [17]. Inulin is a natural food ingredient that is not digested and may stimulate the growth or metabolic activity of certain beneficial bacteria in the human colon [18]. Intestinal microflora contain β-glucosidases that can hydrolyze soy isoflavones to aglycones and promote their absorption. In addition, inulin ferments in the digestive tract, which is associated with the formation of short-chain fatty acids that provide a number of metabolic benefits, including increased sensitivity to insulin and lower serum glucose and lipid levels in the body, and thus protection against type 2 diabetes itself [18,19]. 

In this study, we used a rat model of diabetes mellitus type 2 to investigate the potential treatment effect of soy isoflavones and soy isoflavones with inulin on renal morphology, plasma glucose, ketone bodies, urea, creatinine and renal aquaporins, organic cation and anion transporters, and fatty acids.

## 2. Results

### 2.1. Histological Evaluation of the Kidney

**Figure 1:**
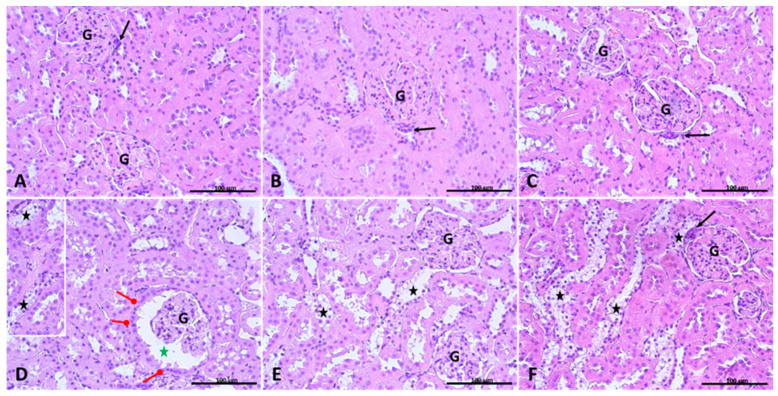
Kidney sections from one representative rat of each group: (**A**) control; (**B**) control supplemented with soy isoflavones; (**C**) control supplemented with soy isoflavones plus inulin; (**D**) with induced diabetes mellitus; (**E**) with induced diabetes mellitus supplemented with soy isoflavones; (**F**) with induced diabetes mellitus supplemented with soy isoflavones plus inulin. Black arrow—macula densa; G—glomerulus; red arrow with round head—Bowman’s capsule with cuboidal epithelium; black star—clear cells in distal tubules; green star—urinary space. H-E ×40. Scale bar: 100 µm.

Histological examination of the kidney of rats from the non-diabetic control group showed a typical arrangement of cortex and medulla with normal renal corpuscles, glomerulus, Bowman’s space, proximal tubules, and distal tubules in both superficial and juxtamedullary nephrons (Figure 1A). Supplementation with soy isoflavones, as well as supplementation with soy isoflavones together with inulin, was associated with an increased area of Bowman’s space in juxtamedullary nephrons compared to the control non-diabetic rats (Figure 1B,C). The kidney of control diabetic rats showed a decreased area of the renal corpuscle, a decreased area of the glomerulus and an increased area of Bowman’s space (Figure 1D). In the kidney of diabetic rats supplemented with soy isoflavones, as well as in the kidney of diabetic rats supplemented with soy isoflavones (Figure 1E) and inulin (Figure 1F), the area of the renal corpuscle and glomerulus appeared to be increased, while the area of Bowman’s space appeared to be decreased compared to control diabetic rats (Figure 1D). In addition, the cytoplasm of many epithelial cells of distal tubules was enlarged and appeared clear by hematoxylin and eosin staining in the kidney of rats of all groups with induced diabetes mellitus. The presence of clear cells in distal tubules was greater in rats with induced diabetes type 2 supplemented with soy isoflavones along with inulin (Figure 1F) compared to control diabetic animals (Figure 1D). Proximal tubule-like epithelium in Bowman’s capsule was observed in part of the renal corpuscles in all diabetic groups (Figure 1D–F), but mainly in the control diabetic group (Figure 1D).

### 2.2. The Percentage of Bowman’s Capsule with Cuboidal Epithelium of the Parietal Cell Layer

**Figure 2:**
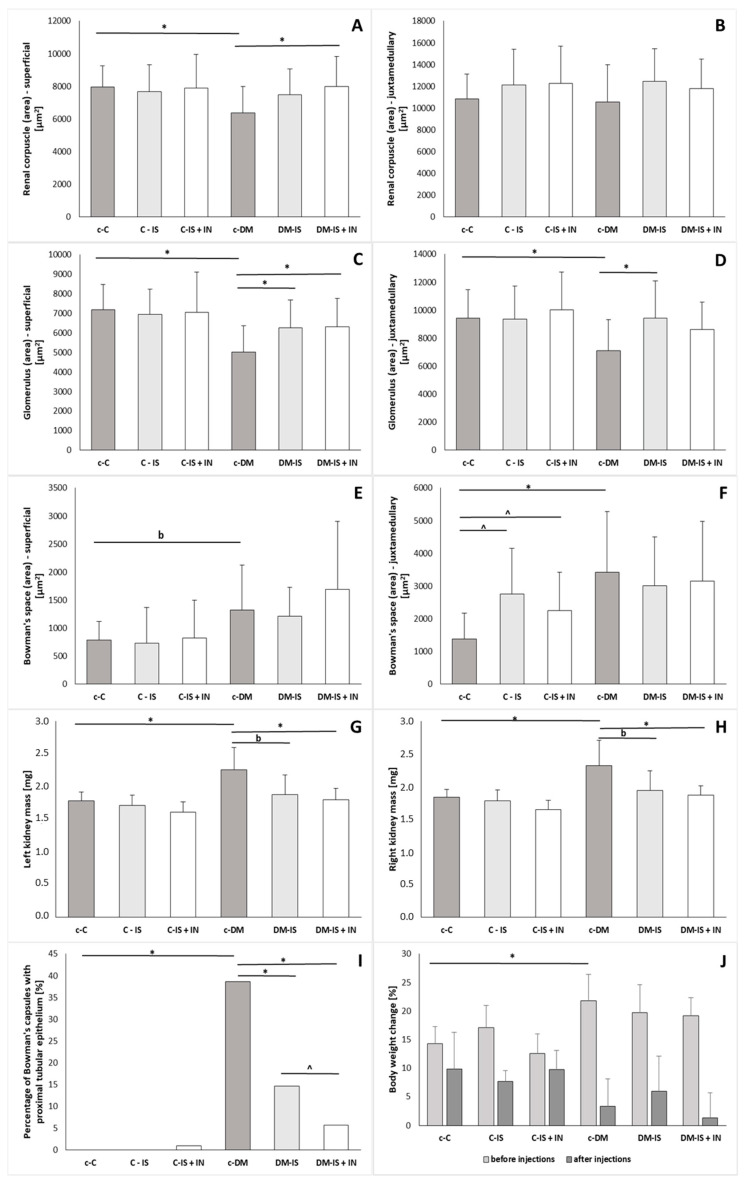
Histogram showing morphometric parameters of nephrons (**A**–**F**); left and right kidney mass (**G**,**H**); percentage of Bowman’s capsules with proximal tubular epithelium (**I**); change in body weight before and after injections (**J**) in each subgroup of rats: c-C (control); C-IS (control supplemented with soy isoflavones); C-IS+IN (control supplemented with soy isoflavones plus inulin); c-DM (with induced diabetes mellitus; DM-IS (with induced diabetes mellitus supplemented with soy isoflavones); DM-IS+IN (with induced diabetes mellitus supplemented with soy isoflavones plus inulin). Values are Average (Mean) ± SD. * *p* < 0.05 (c-DM vs. c-C, DM-IS, DM-IS+IN); ^ *p* < 0.05 (c-C vs. C-IS, C-IS+IN and DM-IS vs. DM-IS+IN); ^b^ *p* < 0.08—at the border of statistically significant difference. (**C**,**G**,**H**,**J**)—parametric Fisher test (Tukey’s post-hoc test); (**A**,**B**,**D**–**F**)—non-parametric Kruskal–Wallis test (Dunn’s post-hoc test); (**I**)—Chi^2^ test (comparison of two proportions).

In the non-diabetic control group and in the non-diabetic control group supplemented with soy isoflavones, no cuboidal epithelium was observed in the parietal layer of Bowman’s capsule. In the control group supplemented with soy isoflavones together with inulin, such epithelium was observed in 1% of the observed renal corpuscles. Bowman’s capsule with proximal tubule-like epithelium in Bowman’s capsule was present in all groups with induced diabetes type 2. The percentage of such epithelium in the diabetic control group (38.67%) was significantly increased compared to the non-diabetic control group (0%). Supplementation with soy isoflavones caused a significant decrease in the percentage of cuboidal epithelium in the diabetic group compared to the control diabetic group (14.67% vs. 38.67%). Supplementation with soy isoflavones together with inulin was associated with a decrease in the percentage of cuboidal epithelium in the diabetic group compared to the control diabetic group (5.67% vs. 38.67%) as well as compared to the diabetic group supplemented with soy isoflavones (14.67% vs. 5.67%) (Figure 2).

### 2.3. Morphometric Analysis

The study revealed significant changes in nephron morphometric parameters (Figure 2; Table S1). The area of the renal corpuscle and glomerulus was statistically decreased, while Bowman’s space was increased (at the border of significance) in superficial nephrons in diabetic control subjects compared to non-diabetic control subjects. Supplementation with soy isoflavones or soy isoflavones with inulin did not cause statistical changes in the non-diabetic group. In the diabetic groups, supplementation with soy isoflavones caused a significant increase in glomerular area compared with the diabetic control group. Supplementation of soy isoflavones with inulin resulted in a statistically significant increase in area of renal corpuscle and glomerulus in the diabetic group. 

In juxtamedullary nephrons, there were no statistically significant differences in the area of renal corpuscles between groups. In the same nephrons, the area of glomerulus was statistically decreased and Bowman’s space was statistically increased in control diabetic rats compared to the control non-diabetic group. Supplementation with soy isoflavones caused a significant increase in glomerular area in the diabetic group and a significant increase in Bowman’s space in the non-diabetic group compared to the corresponding control groups. Supplementation of soy isoflavones with inulin did not cause any significant changes in the morphometry of juxtamedullary nephrons in the diabetic group, while in the non-diabetic group, such supplementation resulted in a statistically increased Bowman’s space compared to the control non-diabetic group (Figure 2; Table S1).

### 2.4. Body Mass, Body Weight Change, Kidney Mass

There were no statistically significant differences in final body mass between groups (Table S2). The comparison of the change in body weight before the injections showed a statistically significant increase in the diabetic control group compared to the non-diabetic control group, and such a tendency was not observed between these groups after the injections. Supplementation with soy isoflavones or soy isoflavones with inulin did not cause any statistically significant differences in the diabetic or non-diabetic groups (Figure 2). The weight of both kidneys (left and right) was significantly increased in the diabetic control group compared to the non-diabetic control group. Supplementation with soy isoflavones or soy isoflavones with inulin did not cause any significant changes in the non-diabetic group. In the diabetic group, supplementation with soy isoflavones resulted in a decrease (at the border of significance) in the weight of both kidneys compared to the control diabetic group. Supplementation of soy isoflavones with inulin caused a significant decrease in the weight of both kidneys compared to the control diabetic group (Table S2).

### 2.5. Water Intake

**Figure 3:**
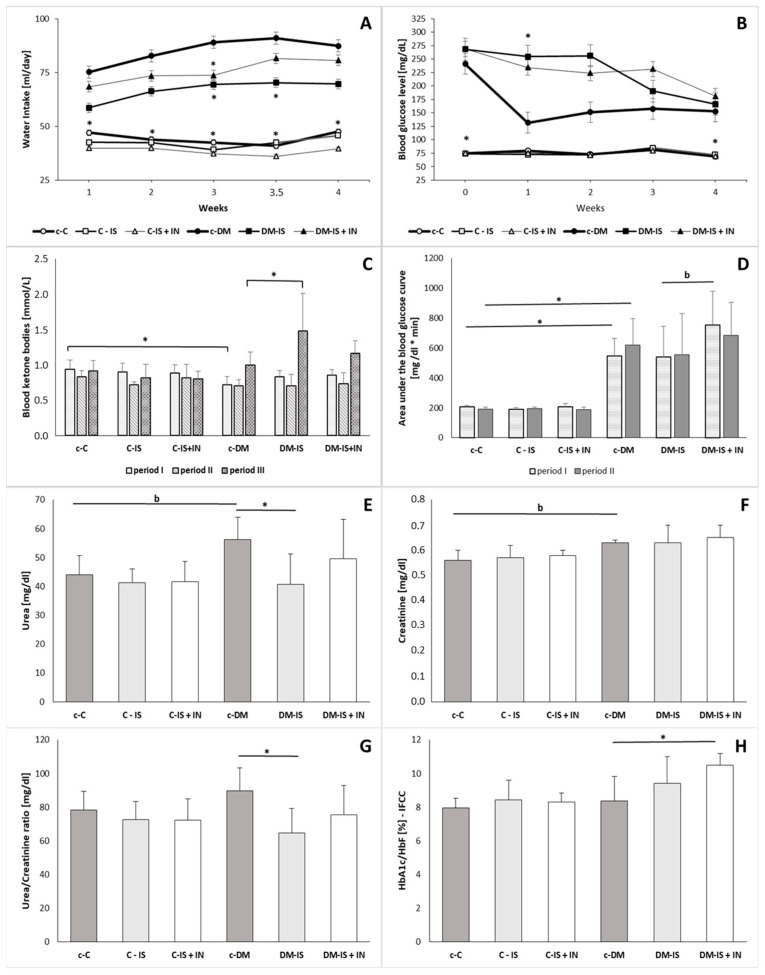
Histograms representing: (**A**) water intake; (**B**) blood glucose level; (**C**) blood ketone bodies level; (**D**) area under the blood glucose curve in groups in the period I and in period II; (**E**) plasma urea; (**F**) plasma creatinine; (**G**) plasma urea/creatinine ratio; (**H**) plasma glycated hemoglobin in groups of rats: c-C (control); C-IS (control supplemented with soy isoflavones); C-IS+IN (control supplemented with soy isoflavones plus inulin); c-DM (with induced diabetes mellitus; DM-IS (with induced diabetes mellitus supplemented with soy isoflavones); DM-IS+IN (with induced diabetes mellitus supplemented with soy isoflavones plus inulin). Values are Average (Mean) ± SD. * *p* < 0.05; ^b^ *p* < 0.08—at the border of statistically significant difference. (**A**–**H**)—parametric Fisher test (Tukey’s post hoc test).

The study showed significant changes in water intake between groups (Figure 3). Water intake was statistically increased in diabetic control rats compared to non-diabetic control rats during all weeks after injections. Supplementation with soy isoflavones or soy isoflavones with inulin did not cause significant changes in water intake in the non-diabetic group. In the diabetic group, supplementation with soy isoflavones caused a significant decrease in water intake 3 weeks and 3.5 weeks after injections. Supplementation of soy isoflavones with inulin caused a significant decrease in water intake 3 weeks after injections.

### 2.6. Blood Glucose, the Area under the Blood Glucose Curve 

There was a significant increase in blood glucose levels immediately after the injections and at the end of the experiment in the diabetic control group compared to the non-diabetic control group. Supplementation with soy isoflavones or soy isoflavones with inulin did not cause significant changes in blood glucose levels. The blood glucose level was significantly increased in the diabetic group after one week of supplementation with soy isoflavones compared to the control diabetic group (Figure 3). 

The area under the blood glucose curve was significantly higher in period I (immediately after the injections) and in period II (4 weeks after the injections) in control diabetic rats compared to control non-diabetic rats. Supplementation with soy isoflavones or soy isoflavones with inulin did not cause significant differences in the non-diabetic group. In the diabetic group, the area under the blood glucose curve was higher (at the border of significance) in period I (immediately after the injections) in the DM-IS+IN group compared with the DM-IS group. After 4 weeks of supplementation, such a difference between the diabetic group supplemented with soy isoflavones and the diabetic group double supplemented with soy isoflavones and inulin was eliminated (Figure 3).

### 2.7. Blood Ketone Bodies

The level of blood ketone bodies was significantly decreased in period I (immediately after injections) in the diabetic control group compared to the non-diabetic control group. Supplementation with soy isoflavones or soy isoflavones with inulin did not cause any significant changes in the non-diabetic group, while in the diabetic group, supplementation with soy isoflavones caused a significant increase in the level of blood ketone bodies at the end of the experiment (period III) compared with the diabetic control group (Figure 3).

### 2.8. Plasma Urea, Creatinine, Glycated Hemoglobin, and Urea/Creatinine Ratio

The results of plasma urea, creatinine, glycated hemoglobin, and urea/creatinine ratio are shown in Figure 3 and Table S2. The comparison of urea and creatinine showed an increase (on the border of a statistically significant difference) in the diabetic control group (c-DM) compared to the non-diabetic control group (c-C). There were no significant changes in the levels of urea/creatinine ratio or HbA1c/HbF between the c-DM and c-C groups. Supplementation with soy isoflavones or soy isoflavones with inulin did not cause significant changes in the non-diabetic group, while in the diabetic group, supplementation with soy isoflavones caused a significant decrease in urea level and urea/creatinine ratio compared to the c-DM group. Supplementation of soy isoflavones with inulin did not cause significant changes in urea, creatinine or urea/creatinine ratio compared to the c-DM group. The level of HbA1c/HbF was significantly increased in the diabetic group supplemented with soy isoflavones with inulin compared to the c-DM group. 

### 2.9. Analysis of Immunohistochemistry (IHC)

#### 2.9.1. Aqp1 Expression

**Figure 4:**
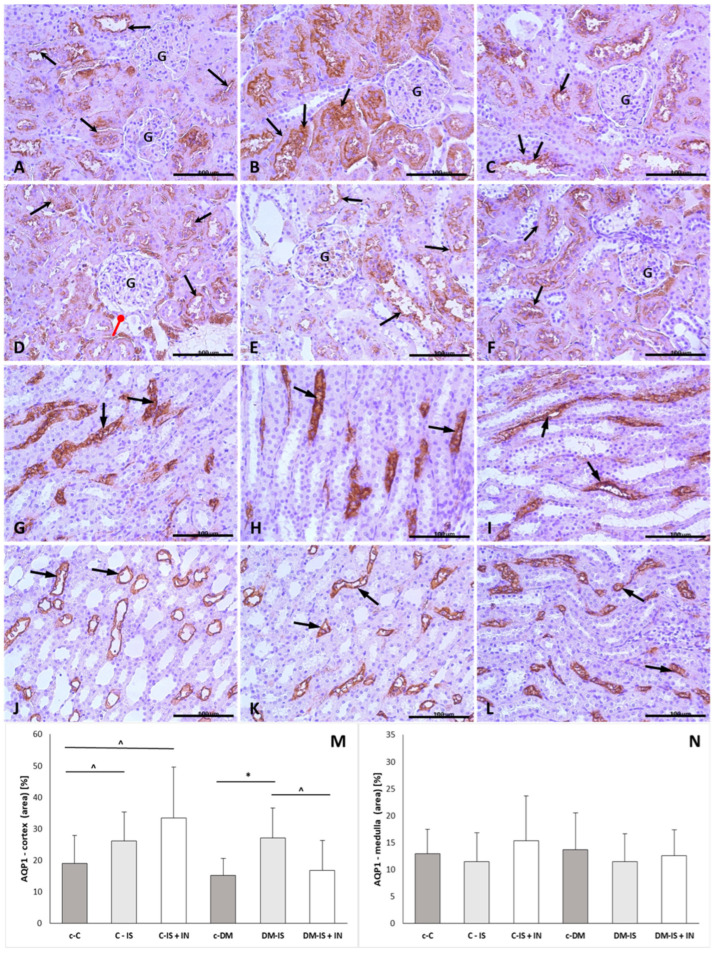
Immunoexpression of AQP1 in kidney sections (cortex: **A**–**F** and medulla: **G**–**L**) of one representative rat from each group: (**A**,**G**) control (c-C); (**B**,**H**) control supplemented with soy isoflavones (C-IS); (**C**,**I**) control supplemented with soy isoflavones plus inulin (C-IS+IN); (**D**,**J**) with induced diabetes mellitus (c-DM); (**E**,**K**) with induced diabetes mellitus supplemented with soy isoflavones (DM-IS); (**F**,**L**) with induced diabetes mellitus supplemented with soy isoflavones plus inulin (DM-IS+IN). Black arrows indicate immunoexpression; red arrow with round head indicates Bowman’s capsule with cuboidal epithelium; G—glomerulus; (**M**)—immunoexpression of AQP1 in the renal cortex; (**N**)—immunoexpression of AQP1 in the renal medulla; (**M**,**N**)—non-parametric Kruskal–Wallis test (Dunn’s post-hoc test); values correspond to Average (Mean) ± SD. * *p* < 0.05 (c-DM vs. DM-IS); ^ *p* < 0.05 (c-C vs. C-IS, C-IS+IN and DM-IS vs. DM-IS+IN); magnification: (**A**–**L**) 40×. Scale bar: 100 µm.

The positive reaction of Aqp1 was visible in the apical and basolateral plasma membrane of the proximal tubule, descending limbs of Henle, and vasa recta in all groups (Figure 4). There were no significant differences in the expression of Aqp1 in the renal medulla between groups. In the cortex, the immunoexpression of Aqp1 was significantly increased in the soy isoflavone-supplemented groups in both diabetic and non-diabetic groups. Supplementation of soy isoflavones with inulin resulted in a significant increase in the non-diabetic group compared to the non-diabetic control group, whereas in the diabetic group, supplementation with soy isoflavones and inulin resulted in a significant decrease in Aqp1 expression compared to the diabetic group supplemented with soy isoflavones (Figure 4; Table S3). 

#### 2.9.2. Aqp2 Expression

**Figure 5:**
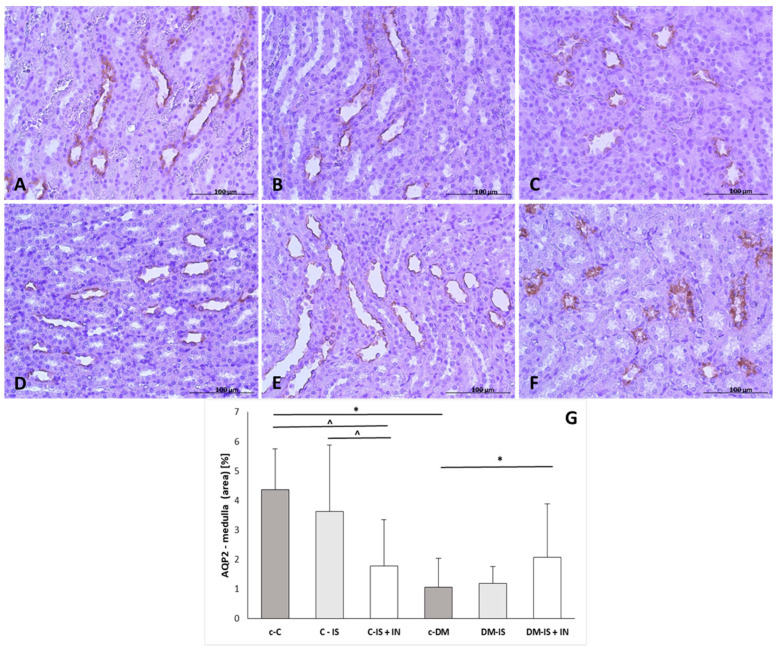
Immunoexpression of AQP2 in kidney sections from one representative rat of each group: (**A**) control (c-C); (**B**) control supplemented with soy isoflavones (C-IS); (**C**) control supplemented with soy isoflavones plus inulin (C-IS+IN); (**D**) with induced diabetes mellitus (c-DM); (**E**) with induced diabetes mellitus supplemented with soy isoflavones (DM-IS); (**F**) with induced diabetes mellitus supplemented with soy isoflavones plus inulin (DM-IS+IN); (**G**)—immunoexpression of AQP2 in the renal medulla; (**G**)—non-parametric Kruskal–Wallis test (Dunn’s post-hoc test); values correspond to Average (Mean) ± SD. * *p* < 0.05 (c-DM vs. c-C, DM-IS+IN); ^ *p* < 0.05 (c-C vs. C-IS+IN and C-IS vs. C-IS+IN); magnification: (**A**–**F**) 40×. Scale bar: 100 µm.

The expression of Aqp2 was present in the distal tubules and in the apical membrane of cells in the collecting tubules and ducts (Figure 5). Immunoexpression of Aqp2 was significantly decreased in both the renal cortex and medulla in the diabetic control group compared to the non-diabetic control group. Supplementation with soy isoflavones caused a significant decrease in the expression of Aqp2 only in the renal cortex and only in the non-diabetic group. Supplementation with soy isoflavones with inulin caused a significant decrease in both cortex and medulla in the non-diabetic group, whereas in the diabetic group, supplementation with soy isoflavones with inulin caused a significant decrease in the renal cortex and a significant increase in the medulla compared to the control diabetic group (Table S3).

#### 2.9.3. AVPR2 Expression

**Figure 6:**
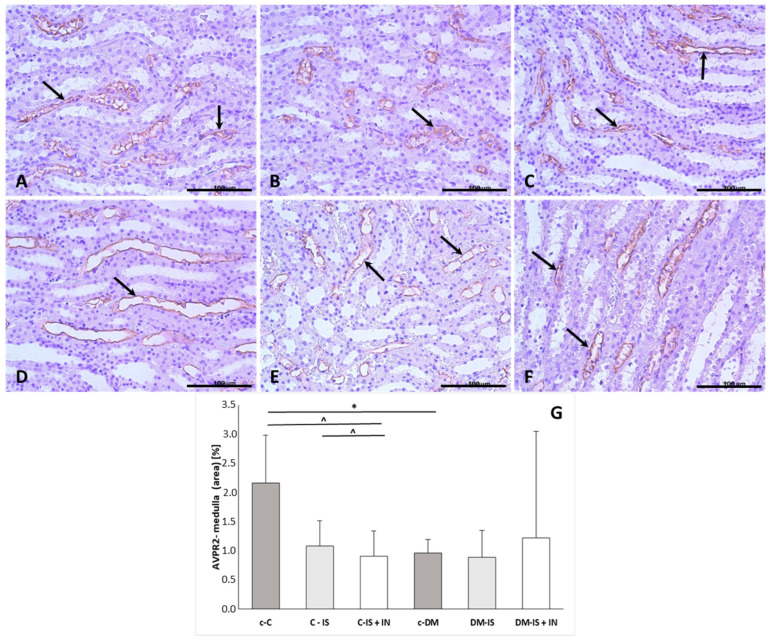
Immunoexpression of AVPR2 in kidney sections from one representative rat of each group: (**A**) control (c-C); (**B**) control supplemented with soy isoflavones (C-IS); (**C**) control supplemented with soy isoflavones plus inulin (C-IS+IN); (**D**) with induced diabetes mellitus (c-DM); (**E**) with induced diabetes mellitus supplemented with soy isoflavones (DM-IS); (**F**) with induced diabetes mellitus supplemented with soy isoflavones plus inulin (DM-IS+IN). Black arrows indicate immunoexpression in the medulla; (**G**)—immunoexpression of AVPR2 in the renal medulla; values correspond to Average (Mean) ± SD. * *p* < 0.05 (c-DM vs. c-C); ^ *p* < 0.05 (c-C vs. C-IS+IN and C-IS vs. C-IS+IN); (**G**)—non-parametric Kruskal–Wallis test (Dunn’s post hoc test); magnification: (**A**–**F**) 40×. Scale bar: 100 µm.

The expression of AVPR2 was mainly visible in the cells of collecting tubules and ducts (Figure 6). The immunoexpression of AVPR2 was significantly decreased in the medulla of the diabetic control group compared to the non-diabetic control group. Supplementation with soy isoflavones or soy isoflavones with inulin did not cause any significant changes in the diabetic group, while in the control group, supplementation with soy isoflavones and supplementation with soy isoflavones with inulin caused a significant decrease compared to the control non-diabetic group (Table S3).

#### 2.9.4. OAT2 (SLC22A7) Expression

**Figure 7:**
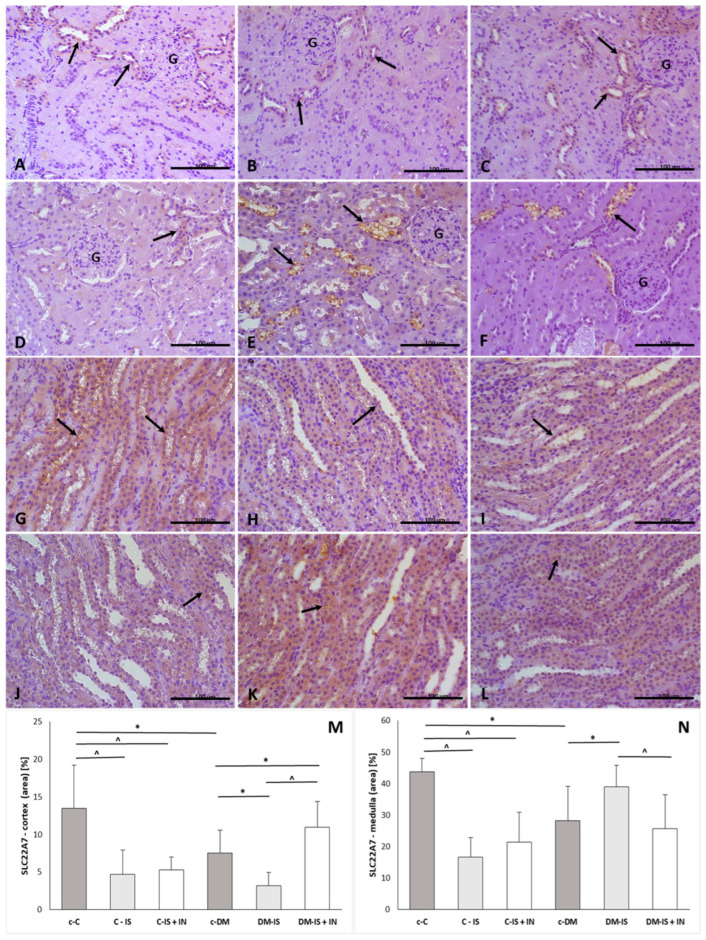
Immunoexpression of SLC22A7 (OAT2) in kidney sections (cortex: (**A**–**F**) and medulla: (**G**–**L**)) of a representative rat from each group: (**A**,**G**) control (c-C); (**B**,**H**) control supplemented with soy isoflavones (C-IS); (**C**,**I**) control supplemented with soy isoflavones plus inulin (C-IS+IN); (**D**,**J**) with induced diabetes mellitus (c-DM); (**E**,**K**) with induced diabetes mellitus supplemented with soy isoflavones (DM-IS); and (**F**,**L**) with induced diabetes mellitus supplemented with soy isoflavones plus inulin (DM-IS+IN). Black arrows indicate immunoexpression in the medulla; G—glomerulus; (**M**)—immunoexpression of SLC22A7 in the renal cortex; (**N**)—immunoexpression of SLC22A7 in the renal medulla; values correspond to Average (Mean) ± SD. * *p* < 0.05 (c-DM vs. c-C, DM-IS, DM-IS+IN); ^ *p* < 0.05 (c-C vs. C-IS, C-IS+IN and DM-IS vs. DM-IS+IN); (**M**)—parametric Fisher test (Tukey’s post-hoc test); (**N**)—non-parametric Kruskal–Wallis test (Dunn’s post-hoc test); magnification: (**A**–**F**) 40×. Scale bar: 100 µm.

The expression of SLC22A7 was present in both the renal cortex and medulla (Figure 7). The immunoexpression of SLC22A7 was significantly decreased in both the cortex and medulla in the diabetic control group compared to the non-diabetic control group. Supplementation with soy isoflavones caused a significant decrease in the cortex and medulla of the non-diabetic group and in the cortex of the diabetic group, while the expression of SLC22A7 was significantly increased in the medulla of the diabetic group. In addition, the immunoexpression of SLC22A7 was significantly increased in the cortex and significantly decreased in the medulla in the diabetic group compared to the diabetic group supplemented with soy isoflavones (Table S3).

#### 2.9.5. Acetyl CoA Expression

**Figure 8:**
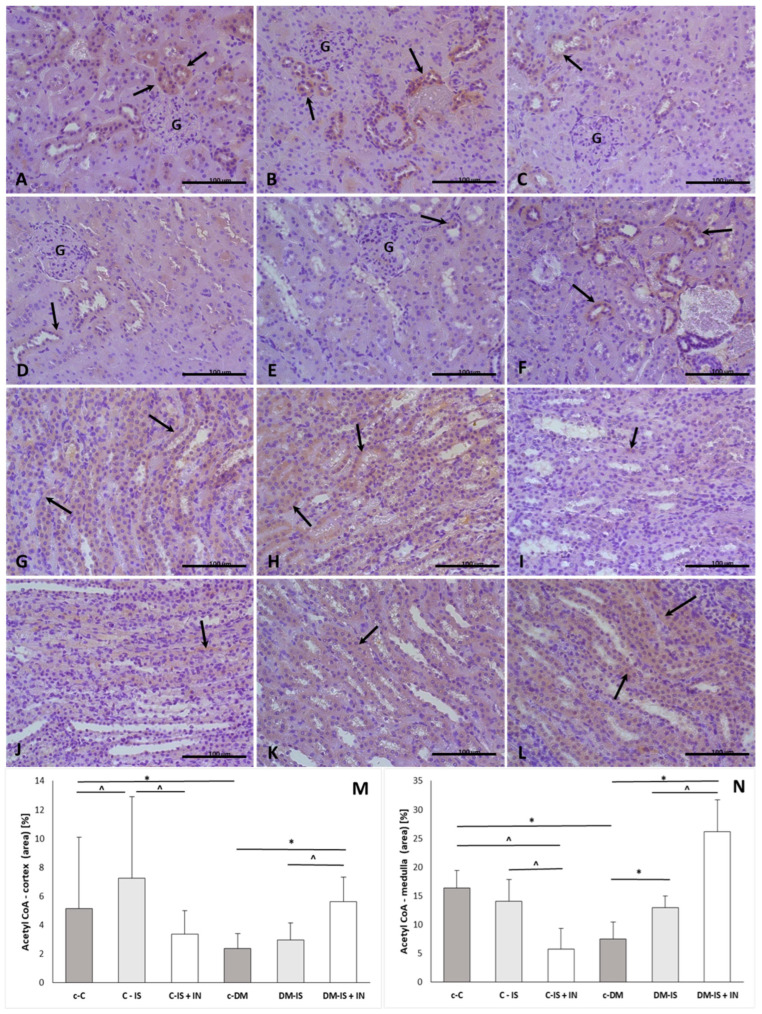
Immunoexpression of ACC-alpha in kidney sections (cortex: (**A**–**F**) and medulla: (**G**–**L**)) of one representative rat from each group: (**A**,**G**) control (c-C); (**B**,**H**) control supplemented with soy isoflavones (C-IS); (**C**,**I**) control supplemented with soy isoflavones plus inulin (C-IS+IN); (**D**,**J**) with induced diabetes mellitus (c-DM); (**E**,**K**) with induced diabetes mellitus supplemented with soy isoflavones (DM-IS); (**F**,**L**) with induced diabetes mellitus supplemented with soy isoflavones plus inulin (DM-IS+IN). Black arrows indicate immunoexpression in the medulla; G—glomerulus; (**M**)—immunoexpression of AcetylCoA in the renal cortex; (**N**)—immunoexpression of AcetylCoA in the renal medulla; values correspond to Average (Mean) ± SD. * *p* < 0.05 (c-DM vs. c-C, DM-IS, DM-IS+IN); ^ *p* < 0.05 (c-C vs. C-IS, C-IS+IN and C-IS vs. C-IS+IN and DM-IS vs. DM-IS+IN); (**M**,**N**)—nonparametric Kruskal–Wallis test (Dunn’s post hoc test); magnification: (**A**–**F**) 40×. Scale bar: 100 µm.

The expression of acetyl CoA was present in both the renal cortex and medulla (Figure 8). The immunoexpression of acetyl CoA was significantly decreased in both the cortex and medulla in the diabetic control group compared to the non-diabetic control group. Supplementation with soy isoflavones caused a significant increase in the expression of acetyl CoA in the cortex in the non-diabetic group and in the medulla in the diabetic group. Supplementation of soy isoflavones with inulin caused a significant decrease in the expression of acetyl CoA in the medulla in the non-diabetic group compared with the non-diabetic control group and a significant increase in the cortex and medulla in the diabetic group compared with the diabetic control group. In addition, the immunoexpression of acetyl CoA was significantly decreased in the non-diabetic group supplemented with soy isoflavones together with inulin compared to the non-diabetic group supplemented with soy isoflavones. In the diabetic group supplemented with soy isoflavones together with inulin, the immunoexpression of acetyl CoA was significantly increased in both cortex and medulla compared with the diabetic group supplemented with soy isoflavones alone (Table S3).

#### 2.9.6. SREBP-1 Expression

**Figure 9:**
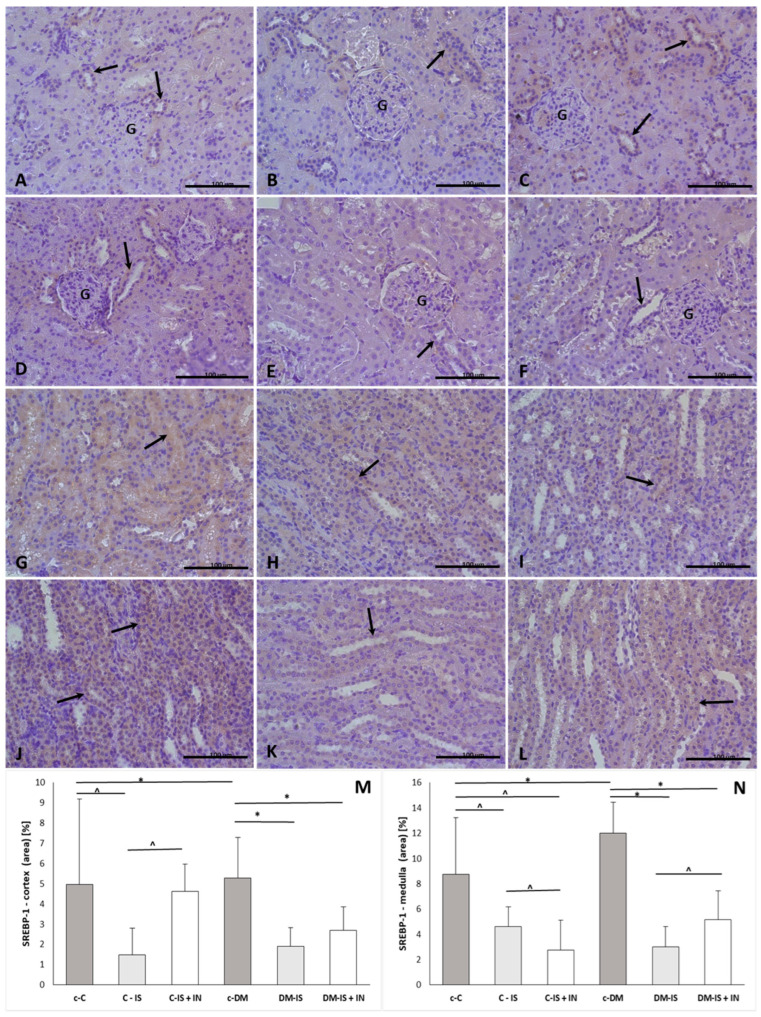
Immunoexpression of SREBP1 in kidney sections (cortex: (**A**–**F**) and medulla: (**G**–**L**)) of one representative rat from each group: (**A**,**G**) control (c-C); (**B**,**H**) control supplemented with soy isoflavones (C-IS); (**C**,**I**) control supplemented with soy isoflavones plus inulin (C-IS+IN); (**D**,**J**) with induced diabetes mellitus (c-DM); (**E**,**K**) with induced diabetes mellitus supplemented with soy isoflavones (DM-IS); (**F**,**L**) with induced diabetes mellitus supplemented with soy isoflavones plus inulin (DM-IS+IN). Black arrows indicate immunoexpression in the medulla; G—glomerulus; (**M**)—immunoexpression of SREBP1 in the renal cortex; (**N**)—immunoexpression of SREBP1 in the renal medulla; values correspond to Average (Mean) ± SD. * *p* < 0.05 (c-DM vs. c-C, DM-IS, DM-IS+IN); ^ *p* < 0.05 (c-C vs. C-IS, C-IS+IN and C-IS vs. C-IS+IN and DM-IS vs. DM-IS+IN); (**M**,**N**)—nonparametric Kruskal–Wallis test (Dunn’s post hoc test); magnification: (**A**–**F**) 40×. Scale bar: 100 µm.

The positive reaction of SREBP-1 was present in both the renal cortex and medulla (Figure 9). The immunoexpression of SREBP-1 was significantly increased in both the cortex and medulla in the diabetic control group compared to the non-diabetic control group. Supplementation with soy isoflavones caused a significant decrease in the expression of SREBP-1 in both the cortex and medulla in both non-diabetic and diabetic groups. Supplementation of soy isoflavones with inulin caused a significant decrease in the cortex and medulla in the diabetic group compared with the control diabetic group, and in the medulla in the non-diabetic group compared with the control non-diabetic group. In addition, the immunoexpression of SREBP-1 was significantly increased in the cortex and significantly decreased in the medulla in the non-diabetic group compared with the non-diabetic group supplemented with soy isoflavones. In the diabetic group, the immunoexpression of SREBP-1 was significantly increased in the medulla in the group supplemented with soy isoflavones with inulin compared to the diabetic group supplemented with soy isoflavones alone (Table S3). 

### 2.10. Fatty Acid Profile

**Figure 10:**
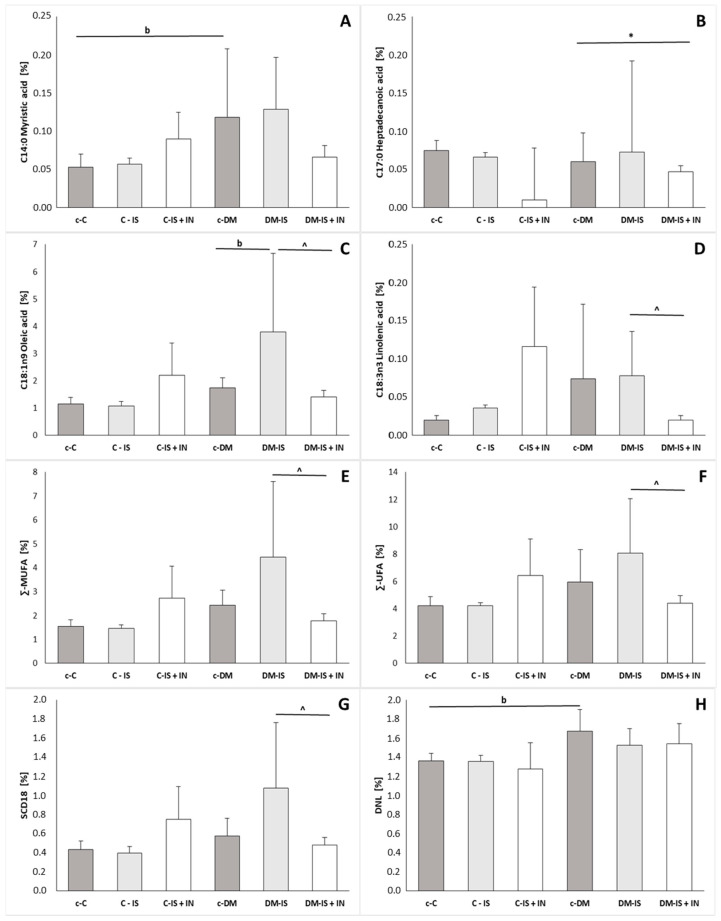
Histograms representing the renal fatty acids: (**A**) C14:0 myristic acid; (**B**) C17:0 heptadecanoic acid; (**C**) C18:1n9 oleic acid; (**D**) C18:3n3 linolenic acid; (**E**) total MUFA (monounsaturated fatty acids); (**F**) total UFA (unsaturated fatty acids); (**G**) SCD18 index stearoyl-CoA desaturase; (**H**) DNL index (de novo lipogenesis) in groups of rats: c-C (control); C-IS (control supplemented with soy isoflavones); C-IS+IN (control supplemented with soy isoflavones plus inulin); c-DM (with induced diabetes mellitus; DM-IS (with induced diabetes mellitus supplemented with soy isoflavones); DM-IS+IN (with induced diabetes mellitus supplemented with soy isoflavones plus inulin). Values are Average (Mean) ± SD. * *p* < 0.05 (c-DM vs DM-IS+IN); ^ *p* < 0.05 (DM-IS vs DM-IS+IN); ^b^ *p* < 0.08—at the border of statistically significant difference. (**C**,**E**–**H**)—parametric Fisher test (Tukey’s post-hoc test); (**A**,**B**,**D**)—non-parametric Kruskal–Wallis test (Dunn’s post-hoc test).

**Figure 11:**
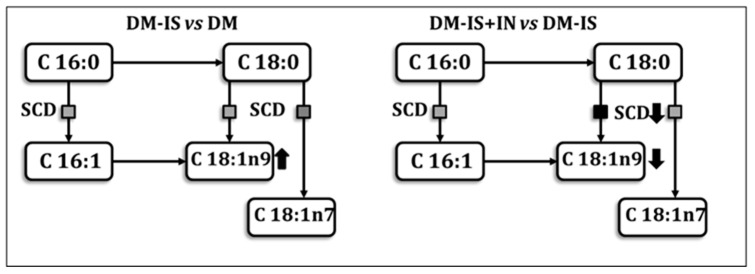
Changes in SCD activity associated with soy isoflavone supplementation and supplementation of soy isoflavones together with inulin.

The study showed significant changes in the fatty acids of the kidney (Figure 10; Table S4). The comparison between the non-diabetic control group (c-C) and the diabetic control group (c-DM) showed an increase (on the border of statistical significance) in the C14:0 (myristic acid) and DNL index in the c-DM group. Supplementation with soy isoflavones or soy isoflavones with inulin did not cause significant changes in the non-diabetic group. In the diabetic group, supplementation with soy isoflavones caused an increase (on the border of significance) in C18:1n9 (oleic acid) compared to the c-DM group. Supplementation of soy isoflavones with inulin caused a significant decrease in C17:0 (heptadecanoic acid) compared to the c-DM group and a significant decrease in C18:1n9 (oleic acid), C18:3n3 (linolenic acid), total MUFA, total UFA and SCD-18 compared to the diabetic group supplemented with soy isoflavones (DM-IS). Changes in SCD activity associated with soy isoflavone supplementation and soy isoflavone supplementation together with inulin are shown in Figure 11.

## 3. Discussion

The present study provides a characterization of the kidney (and related parameters) of rats with (and without) induced diabetes type 2 supplemented with soy isoflavones or soy isoflavones with inulin. 

In the present study, there were no significant differences in final body mass between the groups. A much better indicator is the % change in body mass before the injections (also before supplementation with soy isoflavones and inulin) and after the injections. The change in body mass was significantly higher in rats on a high-fat diet (later diabetic control group) than in rats on a standard diet (later non-diabetic control group), which is not surprising. There were no significant differences in the change of body mass after injections between groups, but some trends may be visible. The change in body mass in control rats with induced type 2 diabetes was decreased compared to control non-diabetic rats, so the opposite situation existed before the injections. The control rats constantly increased their body weight, whereas in the rats with induced diabetes, there were periods (up to 2 weeks after STZ injection) when the body weight decreased and later increased, but not as much as in the control animals. These results are consistent with the work of other researchers [20] and may be associated with decreased glucose metabolism and increased lipid metabolism in animals with induced type 2 diabetes [21]. In the present study, supplementation with soy isoflavones did not cause significant differences, but certain tendencies can be observed. Supplementation with soy isoflavones in the non-diabetic group caused less weight gain than in the control rats after injections, while the group before injections and supplemented with soy isoflavones was characterized by an increased change in body weight compared to the control group. In diabetic animals, the tendency was the opposite, because supplementation with soy isoflavones caused an increase in the change in body weight compared to control non-diabetic animals. Our results are in agreement with the literature. Soy isoflavones, especially at lower doses and shorter intervention periods, are effective in the prevention of obesity [22] in the non-diabetic state. Jee-Youn Shim et al. reported that supplementation with soy isoflavones was associated with significantly greater weight gain than in diabetic control groups [23].

In the current study, the weight of both kidneys was significantly increased in rats with induced type 2 diabetes compared with non-diabetic controls, which is consistent with other data [23,24]. Supplementation with soy isoflavones and supplementation with soy isoflavones and inulin were associated with a reduction in kidney mass compared to control diabetic animals, which is also consistent with reports found in the literature. Jee-Youn Shim et al. reported that all their isoflavone-supplemented groups tended to have lower mean kidney weights than the diabetic control group [23]. The reduction in kidney weight after treatment with soy isoflavones may be explained by the ability of soy isoflavones to reduce the accumulation of fat in the kidney due to the previously documented antilipogenic effect of isoflavones [25]. The results of the current study support the possibility that soy isoflavones and soy isoflavones with inulin may be effective in preventing renal enlargement in diabetic animals. 

In the current study, morphometric analysis of the kidney showed a decreased area of the renal corpuscles and glomerulus and an increased area of Bowman’s space in nephrons of rats with induced type 2 diabetes compared to control non-diabetic rats. This observation is of some interest because there are different (sometimes contradictory) results in the literature. There are reports suggesting that patients (or animals) with type 1 diabetes tend to have larger glomerular volumes, while patients/animals with type 2 diabetes tend to have smaller glomerular volumes [26,27], which explains that increased glomerular volume in patients with type 1 diabetes is associated with glomerular hyperfiltration, absent in patients with type 2 diabetes, and therefore glomerular volume is decreased (or unchanged) in patients with type 2 diabetes [28]. Other studies suggest the opposite, that mean glomerular volume values are much higher in type 2 diabetics than in type 1 diabetics [29]. In the present study, type 2 diabetes was induced by multiple low-dose injections of STZ followed by a high-fat diet. Reports of stereological quantification of renal corpuscles in rats on a high-fat diet showed a decrease in the mean glomerulus volume and the volume fraction ratio of all glomeruli to the renal cortex, and an increase in the mean volume of Bowman’s space compared with control rats [30], which is consistent with our results and can be explained by the fact that HFD induced glomerular atrophy. Perhaps the opposite results found in the literature on renal corpuscle volume are mostly dependent on the type of diet and not on the type of diabetes. Supplementation with soy isoflavones in rats with induced type 2 diabetes was associated with a significant increase in glomerular area in both superficial and juxtamedullary nephrons compared with control diabetic rats. Rats with induced type 2 diabetes supplemented with soy isoflavones together with inulin were characterized by significantly increased renal corpuscle area and increased glomerulus area in superficial nephrons compared to control diabetic animals. This may suggest that soy isoflavones or soy isoflavones with inulin may have reduced glomerular atrophy in rats with induced type 2 diabetes.

The renal corpuscle consists of a glomerulus and the surrounding Bowman’s capsule (visceral and parietal layers). The parietal layer of Bowman’s capsule is typically a layer of simple squamous epithelium, but a high cuboidal epithelium structurally similar to the proximal tubular epithelium has also been reported in human, rat, and mouse kidney [31]. Studies in mice indicate that the presence of cuboidal epithelium in Bowman’s capsule appears to be influenced by sex and age and is regulated by circulating testosterone levels [32] and may be associated with hypertension [31]. Bowman’s capsules with a proximal tubule-like cell layer have been observed in patients with diabetes, lupus, acute glomerular nephritis, and acute tubular necrosis [33]. Haensly et al. discovered that tubular metaplasia in Bowman’s capsule can lead to reabsorption of filtered glucose and sodium in the renal corpuscle, which in turn can affect their concentrations in the proximal and distal tubular lumen, altering blood glucose and GFR [31].

Histologic evaluation of the kidney in the current study revealed that the cytoplasm of many epithelial cells of the distal tubules was enlarged and appeared clear by hematoxylin and eosin staining in the kidney of rats from all groups with induced diabetes mellitus. It is consistent with the literature that in diabetic rats, morphologic and metabolic changes mainly affect the renal distal tubular epithelium and are associated with the development of glycogen-storing clear cell tubules (CCTs) [34,35]. Altered glucose metabolism in CCTs includes upregulation of glycogen synthesis and glycolysis and downregulation of glycogenolysis and gluconeogenesis, and these changes lead to glycogen accumulation [34]. These cells are very similar to Armanni–Ebstein lesions (AELs), which are glycogenotic tubules (with cytoplasm filled with glycogen) of the human diabetic kidney [36]. Long-term experiments in diabetic rats have shown that clear cell tubules eventually evolve into adenomas and carcinomas of clear, acidophilic, or basophilic cell types, representing early preneoplastic changes in rat nephrocarcinogenesis [34]. This early upregulation of protooncogenic pathways is driven in part by the activity of the insulin receptor, which is highly upregulated in rat diabetes-associated CCTs compared to the surrounding tubular apparatus [34,35]. Another metabolic change in the very early stages of nephrocarcinogenesis in the rat and human glycogenotic tubules is de novo lipogenesis [35].

In the current study, water intake was significantly increased in rats with induced type 2 diabetes compared to control non-diabetic rats during all weeks after injection, consistent with previous studies [37]. Polydipsia (an increased thirst) together with polyuria (an increased need to urinate) and polyphagia (an increased appetite) are classic diabetic symptoms, the so-called “3 polys”. Although urine volume was not measured in the current study, we can confirm that rats with induced type 2 diabetes must have had polyuria due to very wet sawdust that had to be replaced more frequently than in non-diabetic animals. Supplementation with soy isoflavones caused a general decrease in water intake (significant 3 and 3.5 weeks after injections) compared to control diabetic rats. These results are also in line with expectations and with the work of other researchers [23]. Supplementation with soy isoflavones and inulin was associated with an overall decrease in water consumption (significant reduction in water intake during the third week after injections) compared to control diabetic rats, but increased water intake (not significant) compared to diabetic rats with induced soy isoflavones. Reduced water intake in diabetic rats supplemented with soy isoflavones and inulin is in agreement with other studies performed in STZ diabetic animals exposed to inulin [38]. 

In the current study, fasting blood glucose levels were significantly elevated in rats with induced diabetes mellitus immediately after injections and 4 weeks (at the end of the experiment) after injections compared with control non-diabetic animals. The area under the blood glucose curve was significantly higher in period I (immediately after the injections) and period II (4 weeks after the injections) in control diabetic rats compared with control nondiabetic rats, while there were no significant changes in the level of glycated hemoglobin between these two groups. In the induced type 2 diabetes group supplemented with soy isoflavones, the fasting glucose level was significantly higher than in control diabetic rats one week after the injections. Although the mean fasting glucose level was lower in control diabetic rats compared to diabetic rats supplemented with soy isoflavones or soy isoflavones with inulin, it is evident that the fasting glucose level decreased continuously over time in both diabetic groups of rats supplemented with soy isoflavones or soy isoflavones with inulin in contrast to control diabetic animals. In addition, the level of glycated hemoglobin was significantly increased in the diabetic group supplemented with soy isoflavones together with inulin compared to the control diabetic group. Glycated hemoglobin represents an average of blood glucose levels over the past 90 days, so this parameter will not show changes (e.g., improvement in glycemia) that have occurred in a relatively short period of time. 

The Oral Glucose Tolerance Test (OGTT) is highly sensitive and specific for the detection of glucose intolerance; however, 2 h blood glucose levels, a criterion for glucose intolerance on the OGTT, may not provide complete information about blood glucose handling after a glucose challenge. The whole glucose study, rather than the plasma glucose levels at a particular point in time, is considered to provide more information about glucose tolerance. The glucose area under the curve, which is an index of the whole glucose excursion after glucose loading, has been widely used to calculate the glycemic index [39]. Analyzing the data on the area under the blood glucose curve and the data on the fasting blood glucose level, a tendency for improvement of glycemia can be observed in rats with induced diabetes type 2 supplemented with soy isoflavones with inulin. This may be related to the fact that soy isoflavones (also with inulin) may have a more pronounced effect on reducing diabetic symptoms by improving the overall metabolism in diabetic animals when the period of supplementation is longer [23].

When glucose is not readily available (and when circulating insulin levels are decreased), lipolysis and the breakdown of fat, which becomes the main source of energy, is promoted, which is associated with the production of ketone bodies. Ketone bodies are mainly produced by the liver (from β-oxidation of fatty acids) and used peripherally as a source of energy. The major ketone bodies are acetoacetate (AcAc) and 3-beta-hydroxybutyrate (3HB), while acetone is the least abundant. The level of ketones, which are always present in the blood, increases during fasting, diets low in digestible carbohydrates, prolonged exercise, and diabetes, which is the most common pathological cause of elevated blood ketones [40,41].

In the current study, the level of blood ketone bodies was significantly decreased immediately after STZ injections and increased (not significantly) at the end of the experiment in control diabetic rats compared with control non-diabetic rats. Supplementation with soy isoflavones was associated with a significantly increased level of blood ketones at the end of the experiment (after 4 weeks of STZ injections) in rats with induced type 2 diabetes compared with control diabetic rats. There are data from the literature indicating that soy protein (in relation to casein) exerts hypochoelsterolemic and hypotriglyceridemic effects in various experimental animals and human subjects [42,43]. The hypocholesterolemic effect of another component of soy—isoflavones—is less obvious [44], whereas the hypotriglyceridemic effect of soy isoflavones is more frequently documented [45]. These effects of soy components may be related (at least in part) to the reduction in cholesterol and triglyceride secretion from the liver into the circulation. Studies using soy proteins have shown that decreased triglyceride secretion in rat liver is a consequence of a selective increase in the production of ketone bodies from exogenous fatty acids [43]. A similar effect to soy proteins was observed in the current experiment in diabetic rats supplemented with soy isoflavones. Additional supplementation with inulin did not cause significant changes in either group.

Results on the expression of aquaporins in strepotoztocin-induced diabetic rats are inconsistent [46,47,48]. In the present study, although the positive reaction of AQP1 was visible in the proximal tubule, descending limb of Henle, and vasa recta in all groups, significant changes in expression between groups were observed only in the cortex but not in the medulla. We did not observe significant differences in the expression of AQP1 between the diabetic control group and the non-diabetic control group, perhaps because the period after the induction of diabetes was too short. Another explanation for the lack of difference in the expression of AQP1 between the diabetic and non-diabetic groups may suggest that AQP1 is not responsible for the defective urinary concentration in diabetic animals [49]. Supplementation with soy isoflavones caused a significant increase in the expression of AQP1 in both the diabetic and non-diabetic groups compared to the respective control groups, which is in agreement with the work of others [50,51]. In the current experiment, the addition of inulin to soy isoflavones in non-diabetic rats was also associated with an increase in AQP1 compared to control non-diabetic rats, and there were no significant differences between the control group supplemented with soy isoflavones alone and the control group supplemented with soy isoflavones together with inulin. However, in the diabetic animals, the addition of inulin seemed to reduce the effect of soy isoflavones. 

In the present study, we found a significantly decreased expression of another aquaporin—AQP2—in the renal medulla of diabetic rats compared to control rats. As with AQP1, the results for AQP2 expression in animals with induced (by STZ) diabetes are contradictory [46,52,53]. Leung et al. reported decreased gene and protein expression of AQP2 and AQP3 in STZ mice with polyuria, which is in agreement with the present study [49]. Downregulation of AQP2 and AQP3 is also common in several types of nephrogenic diabetes insipidus (NDI). 

Expression of water channel protein—AQP2 in the mammalian kidney is mainly regulated by arginine vasopressin (AVP, also called antidiuretic hormone ADH), which regulates water permeability in the renal collecting duct and plays a crucial role in urine concentration and water homeostasis in the body. AVP is mediated by vasopressin receptor subtypes V1a (vascular), V1b (pituitary), and V2 (vascular, renal) [54]. Elevated blood AVP induces protein expression, phosphorylation, and intracellular trafficking of AQP2, resulting in water transport across the epithelium in the collecting ducts [55]. 

In the present study, type 2 diabetes mellitus was induced not only by low doses of STZ but also by HFD. Studies by Kong et al. on mouse kidney biopsies showed that increased urine output in animals on HFD was associated with decreased protein and mRNA levels of AQP2 and decreased mRNA levels of the vasopressin receptor (V2R) [55]. This is consistent with the results of the present study, because not only the expression of AQP2, but also the expression of V2R were decreased in animals with induced type 2 diabetes compared with control animals, which may indicate that a high-fat diet may affect the V2R-AQP2 regulatory pathway. 

In the present study, supplementation with soy isoflavones caused a significant decrease in the expression of AVPR2 in the renal medulla in non-diabetic animals, while in the diabetic group, supplementation with soy isoflavones did not cause significant differences. It is difficult to find information about the influence of soy isoflavones on AQP2 or AVPR2 in the literature, but there is information about other natural compounds such as triterpenoids that have been shown to downregulate renal AQP2 to protect against renal failure [56]. Supplementation with soy isoflavones together with inulin was associated with decreased renal medullary AQP2 expression and decreased renal medullary AVPR2 expression in non-diabetic animals, whereas in rats with induced type 2 diabetes, such supplementation caused a significant increase in renal medullary AQP2 expression compared to diabetic control animals. The observed pattern of changes was not consistent with the results of Michałek et al. [57]. Another parameter measured in this study was sterol regulatory element-binding protein (SREBP), a transcription factor that regulates the synthesis of fatty acids and cholesterol, depending on the isoform. SREBEP-1 preferentially activates genes involved in fatty acid synthesis, together with acetyl-CoA carboxylase and fatty acid synthase (FAS). It is known that the activity of SREBP can be regulated at the transcriptional or post-transcriptional level. Activation of SREBP-1 at the transcriptional level can be upregulated by insulin, glucose, and liver X receptor and downregulated by fatty acids [58].

In the current study, the renal expression of SREBP-1 was significantly increased, whereas the expression of acetyl-CoA (ACC) was significantly decreased in both the cortex and medulla of rats with induced type 2 diabetes compared to controls. Our results seem to be in agreement with the work of Kim et al. [59]. The authors found that inhibition of ACC1 and ACC2 in the liver was associated with increased expression of Srebp-1c, which led to significant activation of Gpat1, the first step in triglyceride synthesis in the liver [59]. Upregulation of Srebp-1 increases expression of genes encoding fatty acid synthesis enzymes such as Acly, Fas, Elovl6, Scd1, Pltp, and Pnpla3, which is also associated with increased levels of circulating triglycerides [60]. In the current experiment, we also observed an increased (although not significant) level of plasma triglycerides in rats with induced type 2 diabetes compared to control non-diabetic animals; data are published in the previous paper [19]. Our results are also consistent with reports in human clinical trials of the hypertriglyceridemic effect of ACC blockade, which may be explained by activation of Gpat1 and incorporation of circulating fatty acids into VLDL triglycerides caused by increased expression of Srebp-1 [59]. 

In the current study, it was also found that de novo lipogenesis (DNL) was increased (at the border of significance) in the kidneys of rats with induced type 2 diabetes compared to control non-diabetic rats. Supplementation with soy isoflavones or soy isoflavones with inulin did not cause any significant changes in either diabetic or non-diabetic group. 

Cytosolic acetyl-CoA is a key substrate in pathways of de novo lipogenesis (DNL) [61]. It can be anticipated that elevated DNL levels should be accompanied by increased ACC concentrations. However, in the current study, SREBP-1 and DNL were both significantly elevated, while ACC was significantly reduced, in the kidneys of rats with induced diabetes type 2. The current results appear to be unexpected, but it is known that acetyl-CoA is produced from citrate by ATP-citrate lyase (ACLY) and from acetate through AcCoA synthase short chain family member 2 (ACSS2). Yenilems et al. reported that upon ACLY depletion, there was an increase in liver DNL, which was also associated with increased expression of nuclear SREBP-1 and of its target DNL enzymes in obese mice. The researchers concluded that in obese mice fed a high-fat diet, hepatic DNL may not be limited by its immediate substrates, acetyl-CoA, or malonyl-CoA. Rather, the limitation may be due to the activities of DNL enzymes [61]. This situation may also be present in the current study in the kidneys of rats with induced diabetes type 2. 

The present study found that supplementation with soy isoflavones was associated with increased ACC1 and decreased SREBP1 in both non-diabetic and diabetic groups compared to corresponding control groups. This is partially in agreement with the work of Huang et al. [62]. The authors have demonstrated that the liver expression of SREBP1 and its downstream target genes (ACC, ACL, and FASN) is suppressed by the supplementation of soy isoflavones in diet-induced obesity rats. [62]. 

The ACC enzyme has two isoforms, ACC1 and ACC2, which are encoded by distinct genes. ACC1 is a cytosolic protein that exhibits high rates of fatty acid synthesis, while ACC2 is located in mitochondria and plays a key role in the regulation of mitochondrial fatty acid beta-oxidation [63]. The regulation of the expression of the ACC gene by dietary soy components may be mediated through different pathways, including thyroid-dependent and SREBP-dependent pathways. The promoter region of the ACC gene contains a thyroid hormone response element, which is under the influence of thyroid hormone [64]. SREBP-1 is downregulated by dietary soy components and it is a transcription factor of the ACC1 gene. Moreover, expression of ACC1 is controlled by two promoters, PI and PII. Dietary soy components significantly decrease only the expression of PI-generated ACC1 mRNA (while PII and PII-generated ACC1 mRNA are not affected) due to inhibition of SREBP-1 binding ability to ACC1 promoter. This indicates that soy components regulate ACC1 mRNA primarily by modulating SREBP-1 binding to PI via nuclear factors other than SREBP-1 itself [63]. This may explain the disparate results observed in the literature regarding the influence of soy components such as isoflavones on the expression of ACC and SREBP-1.

In the current study, supplementation with soy isoflavones in combination with inulin demonstrated a comparable “pattern of influence” on ACC1 and SREBP-1 as the group supplemented solely with soy isoflavones in diabetic animals in comparison to the control diabetic group. A comparison of these parameters between the diabetic group supplemented with soy isoflavones and the diabetic group supplemented with soy isoflavones together with inulin indicates that inulin exerts a modulatory effect. This is evidenced by the observation that in the expression of ACC1, the effect of soy isoflavones appears to be increased, while in the expression of SREBP-1, the effect of soy isoflavones seems to be decreased. The addition of inulin to soy isoflavones in non-diabetic rats also had a modulatory effect on the efficacy of soy isoflavones, as evidenced by a reduction in the expression of ACC1 (in contrast to the diabetic group) following supplementation with soy isoflavones and inulin compared to the group supplemented with soy isoflavones alone. The results from non-diabetic animals are in agreement with the study on oligofructose-fed rats, where the activities of acetyl-CoA carboxylase, fatty acid synthase (FAS), malic enzyme, ATP citrate lyase, and glucose-6-phosphate dehydrogenase were decreased. This lends support to the hypothesis that the administration of inulin/oligofructose may modify the gene expression of lipogenic enzymes [65], although the nature of this modification appears to differ in diabetic and non-diabetic animals. The modulation of lipogenic enzymes by inulin/oligofructose may be explained by the fact that, although they are not digested in the upper part of the digestive tract, they may influence the absorption of macronutrients, delay gastric emptying, and/or shorten the time transit in the small intestine [65].

Fatty acids (FA) serve as a source of energy and also act as signal molecules in various cellular processes. The profile of FA is not solely determined by diet; it also undergoes alterations in the context of specific diseases [66].

Sphingolipids are a class of lipids composed of hydrophobic and hydrophilic regions with different fatty acids. Well-known sphingolipid metabolites include ceramide, sphingosine-1-phosphate (S1P), and ceramide-1-phosphate (C1P). These metabolites have been reported to regulate a number of cellular processes, including cell differentiation, membrane fluidity, anchoring of proteins, immune activation, insulin sensitivity, autophagy, and even cell death [1]. Sas et al. observed elevated levels of long-chain ceramides, including C14:0, C16:0, C18:0, and C20:0, in the kidney cortices of diabetic mice [67]. The current study found that the following fatty acids were elevated in the kidney of rats with induced diabetes type 2: C14:0 (significantly), C16:0, and C18:0 (C20:0 was not measured). These findings are supported by studies that have demonstrated elevated levels of long-chain ceramides in patients with early or overt DKD [1]. Furthermore, the accumulation of ceramides was found to be associated with an increased production of oxygen species in OLEFT rats and mice with DKD following a high-fat diet [68]. The oxidative process plays a significant role in the damage of renal tubular and renal epithelial cells. The current study found that supplementation with soy isoflavones or soy isoflavones with inulin did not result in any notable changes in the previously mentioned renal fatty acids in either group.

The primary enzyme involved in the synthesis of fatty acids, which introduces the initial cis double bond in lipotoxic fatty acids, including stearic acid (C18:0) and palmitic acid (C16:0), is stearyl-CoA desaturase 1 (SCD-1). Such reactions result in the formation of oleic acid (C18:1n9) and palmitoyl acid (C16:1n7), which are less lipotoxic monounsaturated fatty acids (MUFA) [69]. Our analysis of kidney free fatty acids in rats with induced diabetes compared to a control non-diabetic group revealed no significant differences in the levels of C18:0, C18:1n9, C18:1n7, C16:0, and C16:1n7, as well as the activity of SCD-16 and SCD-18. Studies on diabetic mice have demonstrated a reduction in renal SCD-1 expression. However, in clinical settings, SCD-1 expression may not be decreased in all diabetic patients [70]. Conversely, elevated expression of SCD1 is a predominant feature of clear cell renal cell carcinoma (ccRCC). This is evidenced by the early clear cell tubules described above. Consequently, SCD1 has been hypothesized as a potential therapeutic target for ccRCC [71].

It has been suggested that altered intra-renal FFA metabolism may be a contributing factor in the pathogenesis of diabetic nephropathy [70]. One illustrative example is the case of lipolysis enzymes, which have been the subject of intense investigation as a potential therapeutic target for diabetic nephropathy [72]. Another potential therapeutic target is the intra-renal desaturation of SFAs by SCD1. This process may also be involved in the prevention of damage to proximal tubular epithelial cells (PTEC) in diabetic nephropathy [70]. Furthermore, Sieber et al. have indicated that SCD-1 upregulation in diabetic nephropathy may have a protective function against saturated FFA-derived toxic metabolites that are responsible for endoplasmic reticulum stress and podocyte death [73]. Nevertheless, despite the abundance of evidence suggesting that SCD1 activation exerts a protective effect in various metabolic tissues, including kidney cells, there are also indications that inhibiting SCD1 may be beneficial in preventing type 2 diabetes [70]. Therefore, the net effect of inhibiting or activating SCD1 remains uncertain. It is possible that the impact of these processes may vary depending on the specific tissue or organ in question. 

In the current study, the addition of soy isoflavones resulted in a marginal increase in renal C18:1n9 in rats with induced diabetes. Plasma oleic acid may serve as a potential marker of kidney injury [74]. The supplementation of rats with induced diabetes mellitus with both soy isoflavones and inulin resulted in a significant decrease in the level of C18:1n9 and in the activity of SCD-18 compared to rats with induced diabetes and supplemented only with soy isoflavones. This suggests that inulin exerts a modulatory effect on the action of soy isoflavones in rats with induced diabetes mellitus. In vitro studies by Iwai et al. have indicated that decreased SCD1 expressions may be associated with SFA-bound albumin-mediated lipotoxicity and apoptosis in diabetic proximal tubular epithelial cells [70]. The administration of soy isoflavones, either alone or in combination with inulin, did not result in any discernible alterations in rats without induced diabetes mellitus.

The level of SCD-18 in rats with induced diabetes mellitus type 2 and supplemented with soy isoflavones in combination with inulin was found to be lower than in rats with induced diabetes mellitus type 2 and supplemented only with soy isoflavones. However, contrary data exists in the literature regarding the positive or negative effect of this intervention. The regulation of SCD-1 gene expression is influenced by hormones and environmental and dietary factors [75]. Conversely, the levels of total monounsaturated fatty acids (MUFA) and total unsaturated fatty acids (UFA) were significantly decreased in the kidneys of DM-IS+IN rats compared with those of DM-IS rats. Monounsaturated fatty acids are utilized as substrates for the synthesis of phospholipids, triglycerides, and cholesterol esters, and may potentially increase the lipid burden on tissues, which may subsequently lead to insulin resistance [66]. Therefore, a decreased level of MUFA should have a rather positive effect. 

In the current study, there was a marginal increase in the plasma creatinine and plasma urea levels in rats with induced diabetes mellitus type 2 compared to control non-diabetic animals. Creatinine, in conjunction with blood urea nitrogen (BUN), serves as a primary indicator of renal function. Consequently, elevated levels of these parameters indicate a greater degree of kidney damage. [76]. The present study found that supplementation with soy isoflavones or soy isoflavones with inulin did not result in any significant changes in the plasma creatinine level in either the diabetic or non-diabetic group. However, the level of urea was significantly decreased after supplementation with soya isoflavones in rats with induced diabetes type 2. Previous studies have indicated that genistein can result in downregulation of serum creatinine and BUN in mice with induced hyperuricemia [76]. Similarly, the administration of daidzein to rats with streptozotocin-induced diabetes was associated with a significant reduction in creatinine and BUN levels. [11]. 

A multitude of exergonic substances can influence serum creatinine by affecting tubular secretion without affecting the glomerular filtration rate. The active tubular secretion of creatinine is mediated by basolateral uptake by organic anion transporters OAT2 (SLC22A7) and OAT3 and organic cation transporters OCT2 (SLC22A2) and OCT3, and apical efflux by multidrug and toxin extrusion transporters MATE1 and MATE2-K [77]. 

The present study observed a significant decrease in the expression of organic anion transporter 2 (OAT2) in the renal cortex and medulla of control diabetic rats compared to control non-diabetic animals. Supplementation with soy isoflavones resulted in a significant decrease in the expression of OAT2 in the renal cortex and medulla in the non-diabetic group. In contrast, in the diabetic group, a significantly decreased expression was observed in the cortex, while in the medulla, the expression was significantly increased compared to the respective control groups. This finding is consistent with another study [78]. The decreased expression of OAT2 is associated with an increased (borderline significant) level of serum creatinine in rats with induced diabetes mellitus type 2 compared to control non-diabetic animals. The lack of significant changes in the level of creatinine in both diabetic and non-diabetic groups compared to corresponding control groups may be explained by the fact that there are additional creatinine transporters that were not evaluated in the present study and may also be influenced by soy isoflavones or soy isoflavones with inulin. The resulting action of these transporters affects the level of plasma creatinine.

The urea-to-creatinine ratio (UCR) has been proposed as a valuable clinical tool. Serum urea and creatinine levels typically rise in proportion, which is associated with a progressive decline in renal function. Several studies have indicated that UCR is strongly associated with poor clinical outcomes in various conditions, including acute kidney injury (AKI), acute decompensated heart failure, chronic heart failure, acute myocardial infarction, and ischemic stroke [79]. The current study demonstrated a significant reduction in UCR in diabetic rats supplemented with soy isoflavones in comparison to control diabetic rats. This suggests a potential beneficial effect of soy isoflavones in rats with induced diabetes type 2. 

## 4. Materials and Methods

### 4.1. Animal Care and Study Design

The experiment was performed on thirty-six male Sprague–Dawley (SD) rats obtained from Animalab, Teltow, Germany at the age of 9–10 weeks and treated as previously described [19]. Following an acclimatization period, the animals were randomly divided into two equal main groups: a non-diabetic group and a diabetic group. The animals in the non-diabetic group were fed a regular chow, while the diabetic group was fed a high-fat diet (HFD) to induce insulin resistance [19,80,81]. Animals in both groups had ad libitum access to water and food until the end of the experiment. Two months later, rats on the HFD diet received two intraperitoneal injections of streptozotocin (STZ, 30 mg/kg b.w.; Sigma-Aldrich, Saint Louis, MO, USA) three days apart to induce type 2 diabetes [80], while non-diabetic rats were injected twice with 0.25 mL/kg of 0.9% NaCl, with the injections spaced three days apart. Prior to the second injection, blood glucose levels were tested using a glucometer. Rats with a glucose level greater than 16.67 mmol/L were excluded from the second injection. The nondiabetic rats were then randomly divided into three subgroups (n = 6): (i) Control (c-C), (ii) Rats treated with 100 mg/kg b.w. isoflavones (C-IS), and (iii) Rats treated with 100 mg/kg b.w. isoflavones plus 100 mg/kg b.w. inulin (C-IS+IN). The diabetic rats were divided into three parallel subgroups (n = 6): (iv) Control (c-DM), (v) Rats treated with 100 mg/kg b.w. isoflavones (DM-IS), and (vi) Rats treated with 100 mg/kg b.w. isoflavones plus 100 mg/kg b.w. inulin (DM-IS+IN). The isoflavones and inulin were administered orally for 30 days, once per day in the morning, in the form of small pellets of isoflavones or isoflavones plus inulin, pressed into a piece of bread. The rats in both control subgroups (c-C and c-DM) received pellets without any substances. During the experiment, the water consumption of each cage was monitored daily, and the average values for each rat were calculated. The body weight of each rat was measured weekly. Tail blood glucose levels were quantified using an Optium Xido (Abbott, Warszawa, Poland) portable glucose meter after 14 h of fasting. Tail blood ketone levels were determined three times during the experiment after 14 h of fasting. At the conclusion of the experiment, the rats were six months of age. Following a 12–14-h fast, the animals were euthanized under general anesthesia (2 mL/kg b.w.). Blood was collected directly from the heart and analyzed for urea, creatinine, HbA1c/HbF. The kidneys were removed and weighed. One kidney from each rat was fixed in 4% paraformaldehyde and embedded in paraffin for histological staining and immunohistochemical analysis. The second kidney was washed with normal saline and stored at −70 °C until use. 

**Figure 12:**
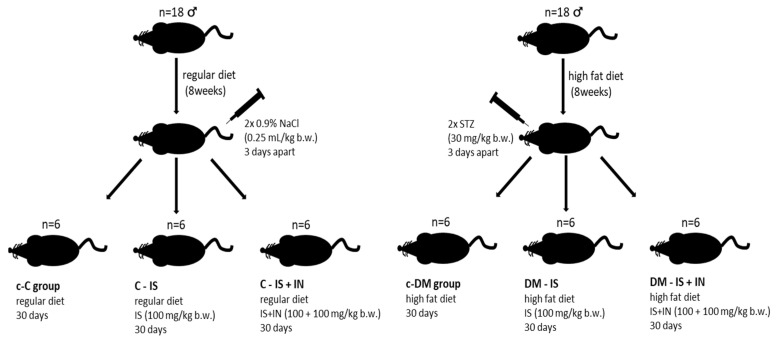
Schema of the experiment.

The experiment was conducted in accordance with Polish law and approved by the Local Ethical Committee on Animal Testing in Poznań, Poland (approval no. 60/2016). Figure 12 depicts the experimental schema.

### 4.2. Oral Glucose Tolerance Test (OGTT)

For the oral glucose tolerance test (OGTT), rats were fasted for 14 h before being orally loaded with glucose (1.0 mg/g body weight). Tail blood glucose levels were measured using an Optium Xido (Abbott) portable glucose meter at 0, 60, and 120 min after glucose administration for plasma glucose levels. The total area under the blood glucose concentration curve (AUC) was calculated by the trapezoidal method from time 0 to 120 min after oral glucose administration. The oral glucose tolerance test was performed twice during the experiment: I. after the second injection with STZ but before supplementation (period I); II. at the end of the experiment (period II).

### 4.3. Morphological Analysis and Immunohistochemistry (IHC)

Following the conclusion of the experiment, kidney sections from all groups of rats were fixed by immersion in 4% paraformaldehyde and embedded in paraffin. For morphological analysis, a series of kidney sections (3–5 μm) were mounted on polylysine slides and stained with hematoxylin and eosin (H-E).

The presence of aquaporins (AQP1, AQP2), organic anion transporter (SLC22A7), the receptor for AVPR2, the receptor for Acetyl CoA (ACC-α), and the receptor for sterol regulatory element-binding protein 1 (SREBP-1) in kidney tissues was identified through immunohistochemical (IHC) reactions with specific primary antibodies.

- Mouse monoclonal anti-AQP1 antibody (Abcam, Cambridge, UK, cat. no. ab9566); 1:100.
- Rabbit monoclonal anti-AQP2 antibody (Abcam, Cambridge, UK, cat. no. ab199975); 1:100.
- Rabbit polyclonal anti-AVPR2 antibody (LSBio an Absolute Biotech Company, Shirley, MA, USA; cat. no. LS-A272); 26 µg/mL.
- SLC22A2 antibody (Abcam; cat. no. ab230629); 1:100.
- Mouse monoclonal anti-ACC-alfa antibody (Santa Cruz Biotechnology, Santa Cruz, CA, USA; cat. co. sc137104); 1:200.
- Mouse monoclonal anti-SREBP-1 antibody (Santa Cruz Biotechnology, Santa Cruz, CA, USA; cat. no. sc13551); 1:200.

The deparaffinized sections were subjected to microwave heating in citrate buffer (pH 6.0) to facilitate epitope retrieval. Following a slow cooling period to room temperature, the slides were washed twice in PBS for 5 min. They were then incubated for 60 min with the previously mentioned primary antibodies. Subsequently, the slides were stained with the avidin–biotin–peroxidase system, utilizing 5,5′-diaminobenzidine (DAB) as the chromogen, in accordance with the established staining protocol (EnVision+System-HRP, code K4010, DakoCytomation, Glostrup, Denmark). The sections were then washed in distilled H₂O and counterstained with hematoxylin, with the exception of those sections exhibiting nuclear localization of receptors. A negative control was employed in which the primary antibody was omitted. Positive staining was defined by the visual identification of DAB brown pigmentation with a light microscope (Leica DM5000B, Leica Microsystems, Wetzlar, Germany).

### 4.4. Morphometry

Following histological processing of the tissue, the slides were evaluated using a light microscope and LAS V4.4 software (Leica DM5000B, Germany). Kidney sections were stained with H-E, and their images were captured by a digital camera under the objective lens magnification of 40×. The following morphometric parameters were measured: renal corpuscle area (μm^2^), glomerular area (μm^2^), and Bowman’s space area (μm^2^). In each animal, 12 renal corpuscles were measured, comprising six from superficial nephrons and six from juxtamedullary nephrons. In total, 36 renal corpuscles from superficial nephrons and 36 renal corpuscles from juxtamedullary nephrons were analyzed from each of the six groups of rats.

### 4.5. The Percentage of Bowman’s Capsule with Cuboidal Epithelium of the Parietal Cell Layer

The number of renal corpuscles in the right and left kidneys of each animal was counted. For each corpuscle, the parietal layer of Bowman’s capsule was classified as squamous (normal) or cuboidal (proximal tubule-like) epithelium. The percentage of Bowman’s capsule with cuboidal epithelium of the parietal cell layer was determined for each animal in each group.

### 4.6. Semi-Quantitative Determination of Protein Expression

Following immunostaining with a specific antibody, 10 poles (viewed at 40× objective magnification) of kidney cortex and 10 poles from the kidney medulla of each animal (for each detected protein) were selected for semi-quantitative determination of protein expression. Semi-quantitative determination of protein expression was performed with the use of ImageJ Fiji (ImageJ 1.54f, Java 1.8.0_322, Wayne Rasband and contributors, National Institutes of Health, Bethesda, MD, USA) according to the Crowe and Yue protocol [82].

### 4.7. Isolation of Fatty Acids

Fatty acids were extracted according to the Folch method [83]. A total of 60 mg of liver tissue was homogenized and saponified with 3 mL of methanol/chloroform (1:2). Samples were then centrifuged, and 1 mL of the resulting supernatant was saponified in a 2 mol/L KOH methanolic solution at 70 °C for 20 min. Thereafter, the samples were methylated with 2 mL of 14% boron trifluoride in methanol under the same conditions. Subsequently, 2 mL of n-hexane and 10 mL of a saturated NaCl solution were added. One milliliter of the n-hexane phase was collected for analysis. 

### 4.8. Analysis of Fatty Acid Methyl Esters

Gas chromatography was conducted using the Agilent Technologies 7890A GC System-Agilent, Santa Clara, CA, USA-(SUPELCOWAX™ 10 Capillary GC Column (15 mm × 0.10 mm, 0.10 μm)). The chromatographic conditions were as follows: the initial temperature was 60 °C for 0 min, increased at a rate of 40 °C/min to 160 °C (0 min), increased at a rate of 30 °C/min to 190 °C (0.5 min), and then increased at a rate of 30 °C/min to 230 °C for 2.6 min, where it was maintained for 4.9 min. The temperature was then increased at a rate of 30 °C/min to 190 °C (0.5 min), and then at a rate of 30 °C/min to 230 °C for 2.6 min, where it was maintained for 4.9 min. The total analysis time was approximately 8 min, with a gas flow rate of 0.8 mL/min and hydrogen serving as the carrier gas. Fatty acids were identified by comparing their retention times with those of commercially available standards.

### 4.9. Statistical Analysis

The statistical analysis was conducted using Statistica version 13.3, with results expressed as means ± standard deviations and median with Q1–Q3. Normality was tested using the Shapiro–Wilk test. For data that exhibited a normal distribution, the Fisher test was employed, and the Tukey test was utilized for post hoc comparisons. Conversely, for data that did not exhibit a normal distribution, the Kruskal–Wallis test was utilized, followed by Dunn’s post hoc test for comparisons between groups. To compare categorical variables, the Chi-square test (comparison of two proportions test performed in MedCalc software) was employed (MedCalc Statistical Software version 16.4.3; Ostend, Belgium). In all cases, a *p*-value of less than 0.05 was considered statistically significant, while a *p*-value of less than 0.08 was considered to indicate a trend toward statistical significance.

## 5. Conclusions

In conclusion, supplementation with soy isoflavones was shown to have a positive effect on plasma urea, plasma urea/creatinine ratio, glycemia, and water intake, as well as the weight, morphology, and function of the kidney in rats with induced diabetes type 2. Furthermore, additional supplementation with inulin has been observed to exert a modulatory effect on the action of soy isoflavones.

## 6. Limitations

The Local Ethical Committee on Animal Testing recommended that six animals be used per subgroup. This is a sufficient but minimal number of animals. 

## Data Availability

The data presented in this study are available on reasonable request from the corresponding author.

## Supplementary Materials

The following supporting information can be downloaded at: https://www.mdpi.com/article/10.3390/ijms25105418/s1.
