# Supplementary material for: The Influence of Soy Isoflavones and Soy Isoflavones with Inulin on Kidney Morphology, Fatty Acids, and Associated Parameters in Rats with and without Induced Diabetes Type 2
Source: Int J Mol Sci. 2024 May 16;25(10):5418. doi: 10.3390/ijms25105418 (PMC11121859; doi:10.3390/ijms25105418)
Supplement: Supplementary file 1 [file ijms-25-05418-s001.zip › ijms-2936575-supplementary.pdf]

Table S1. Morphometric analysis of renal corpuscles in each subgroup of rats.

**Area**	**Control group**			**Diabetes group**		
[μm2]	**c-C**	**C-IS**	**C-IS + IN**	**c-DM**	**DM-IS**	**DM-IS + IN**
**Superficial nephrons**						
**Renal Corpuscle**						
Mean ± SD	7973.36 ± 1277.06	7669.56 ± 1650.42	7889.93 ± 2071.89	6358.02 ± 1619.46	7468.21 ± 1598.69	7999.76 ± 1818.28
Median	7968.28	7527.27	7710.67	6433.71*	7652.93	7526.53$^{\Delta}$
Q1-Q3	7185.37-8714.84	6469.31-8998.82	6549.88-9287.22	4973.71-7472.8	6166.63-8637.23	6784.14-8737.06
**Glomerulus**						
Mean ± SD	7185.7 ± 1298.74	6937.2 ± 1315.56	7066.65 ± 2050.65	5033.39 ± 1346.38*	6251.9 ± 1430.75$^{\Delta}$	6307.95 ± 1462.61$^{\Delta}$
Median	7343.11	7172.44	6963.25	4872.02	5998.2	6088.64
Q1-Q3	6339.22-7826.05	6105.39-8006.68	5671.38-8547.07	4021.56-5800.83	5217.59	5371.16-7313-41
**Bowman's space**						
Mean ± SD	787.66 ± 327.44	732.36 ± 635.57	823.29 ± 672.64	1324.63 ± 806.4	1216.31 ± 514.54	1691.82 ± 1212.91
Median	751.86	588.31	718.43	1273.48^{c}	1126.22	1399.21
Q1-Q3	552.08-976.83	180.51-1153-32	348.66-1115.6	607.06-2082.33	832.11-1640-37	860.04-2072.43
**Juxtamedullary nephrons**						
**Renal Corpuscle**						
Mean ± SD	10831.07 ± 2297.33	12103.74 ± 3299.2	12282.43 ± 3413.32	10541.86 ± 3418.17	12459.62 ± 3004.68	11774.44 ± 2709.86
Median	10839	11402.44	11977.14	10303.19	11673.45	12269.98
Q1-Q3	9483.69-11363.16	9861.78-13367.69	9798.74-13861.23	7572.59-12872.61	10173.18-14461.04	9899.94-13678.78
**Glomerulus**						
Mean ± SD	9446.8 ± 2014.34	9348.6 ± 2383.54	10028.52 ± 2700.08	7109.57 ± 2222.26	9448.63 ± 2646.1	8624.91 ± 1961.
Median	9133.1	8806.44	10034.43	6582.39*	9591.99$^{\Delta}$	8598.31
Q1-Q3	8439.39-10175.16	7478.93-10574.52	8076.18-11371.98	5476.5-8742.64	7454.21-10878.72	7615.79-9911.63
**Bowman's space**						
Mean ± SD	1384.27 ± 790.15	2755.14 ± 1397.3	2253.91 ± 1164.9	3432.28 ± 1852.71	3010.99 ± 1491.9	3149.53 ± 1829.17
Median	1180.81	2488.5*	2013.03*	3114.14*	2857.4	2968.12
Q1-Q3	730.38-1854.07	1886.98-3205.7	1550.31-2526.47	1888.4-4775.87	1752.06-3951.63	1774.2-4139.6

Data are expressed as mean ± SD (standard deviation), M (median), Q1-Q2 (lower and upper quartile) of 6 rats in each group: c-C (control); C-IS (control supplemented with soy isoflavones); C-IS+IN (control supplemented with soy isoflavones plus inulin); c-DM (with induced diabetes mellitus; DM-IS (with induced diabetes mellitus supplemented with soy isoflavones); DM-IS+IN (with induced diabetes mellitus supplemented with soy isoflavones plus inulin). *$p < 0.05$ *vs.* c-C, $^{\Delta}p < 0.05$ *vs.* c-DM, $^{c}P < 0.08$ *vs.* c-C – on the border of statistically significant differences. Significances associated with Mean±SD -

parametric Fisher test (Tukey's post-hoc test); Significances associated with Median - non-parametric Kruskal-Wallis test (Dunn's post-hoc test).

Table S2. Final body mass, weight of kidney (left and right), plasma urea, creatinine, glycated hemoglobin and urea/creatinine ratio.

Parameters	Control Groups			Diabetes Groups		
	c-C	C-IS	C-IS + IN	c-DM	DM-IS	DM-IS + IN
**Body mass** [g]						
Mean ± SD	511.67 ± 40.58	535.83 ± 60.2	504.17 ± 42.48	556.67 ± 34.45	525 ± 47.22	490 ± 37.95
Median	520	545	500	570	530	495
Q1-Q3	490 - 530	470 - 555	480 - 520	530 - 580	470 - 570	460 - 520
**Left kidney mass** [mg]						
Mean ± SD	1.78 ± 0.14	1.71 ± 0.16	1.61 ± 0.16	2.26 ± 0.34*	1.88 ± 0.3^{b}	1.8 ± 0.17$^{\Delta}$
Median	1.75	1.65	1.6	2.17	1.88	1.76
Q1-Q3	1.69 - 1.94	1.62 – 1.79	1.47 – 1.67	1.97 – 2.63	1.59 – 1.94	1.64 – 1.97
**Right kidney mass** [mg]						
Mean ± SD	1.84 ± 0.12	1.78 ± 0.17	1.65 ± 0.14	2.32 ± 0.39*	1.94 ± 0.3^{b}	1.87 ± 0.14$^{\Delta}$
Median	1.79	1.75	1.64	2.21	1.95	1.83
Q1-Q3	1.77 – 1.98	1.68 – 1.9	1.54 – 1.71	2.00 – 2.76	1.67 – 1.97	1.78 – 1.99
**Urea** [mg/dl]						
Mean ± SD	44 ± 6.69	41.33 ± 4.8	41.67 ± 6.95	56.17 ± 7.78^{c}	40.67 ± 10.61$^{\Delta}$	49.5 ± 13.68
Median	40.5	41	41.5	56.5	38	55
Q1-Q3	39 - 52	39 - 46	38 - 42	50 – 64	33 - 42	33 - 57
**Creatinine** [mg/dl]						
Mean ± SD	0.56 ± 0.04	0.57 ± 0.05	0.58 ± 0.02	0.63 ± 0.01^{c}	0.63 ± 0.07	0.65 ± 0.05
Median	0.57	0.57	0.58	0.63	0.65	0.64
Q1-Q3	0.54 – 0.6	0.54 – 0.62	0.57 – 0.59	0.62 – 0.64	0.56 – 0.69	0.61 – 0.68
**Urea/Creatinine ratio**						
Mean ± SD	78.46 ± 11.09	72.53 ± 10.94	72.19 ± 12.82	89.56 ± 13.63	64.8 ± 14.54$^{\Delta}$	75.5 ± 17.29
Median	75.91	69.3	71.27	90.41	63.57	80.58
Q1-Q3	68.85 – 88.33	67.74 – 71.88	64.41 – 74.55	78.13 – 103.17	55.07 – 70.37	54.24 – 90.28
**HbA1c/HbF** (%)-IFCC						
Mean ± SD	7.97 ± 0.55	8.43 ± 1.19	8.32 ± 0.54	8.38 ± 1.44	9.4 ± 1.61	10.48 ± 0.72$^{\Delta}$
Median	7.9	8.15	8.4	7.9	9.65	10.45
Q1-Q3	7.5 – 8.3	7.4 – 9.5	7.7 – 8.7	7.4 – 8.7	7.8 – 10.9	10.1 – 10.9

Data are expressed as mean ± SD (standard deviation), M (median), Q1-Q2 (lower and upper quartile) of 6 rats in each group: c-C (control); C-IS (control supplemented with soy isoflavones); C-IS+IN (control supplemented with soy isoflavones plus inulin); c-DM (with induced diabetes mellitus; DM-IS (with induced diabetes mellitus supplemented with soy isoflavones); DM-IS+IN (with induced diabetes mellitus supplemented with soy isoflavones plus inulin). $^{*}p < 0.05$ *vs.* c-C, $^{\Delta}p < 0.05$ *vs.* c-DM, $^{b}p < 0.08$ *vs.* c-DM and $^{c}p < 0.08$ *vs.* c-C – on the border of statistically significant differences. Significances associated with Mean±SD - parametric Fisher test (Tukey's post-hoc test); Significances associated with Median - non-parametric Kruskal-Wallis test (Dunn's post-hoc test).

Table S3. Semi-quantitative determination of immunoexpression of AQP1, AQP2, AVPR2, SLC22A7, Acetyl CoA, SREBP-1.

**Parameters**	**Control Groups**			**Diabetes Groups**		
area [%]	**c-C**	**C-IS**	**C-IS + IN**	**c-DM**	**DM-IS**	**DM-IS + IN**
**AQP1 - cortex**						
Mean ± SD	19.03 ± 8.9	26.14 ± 9.19	33.42 ± 16.17	15.18 ± 5.52	27.06 ± 9.56	16.74 ± 9.53
Median	18.35	23.17*	30.71*	13.98	27.18$^{\Delta}$	15.24$^{\&}$
Q1-Q3	12.18 – 26.04	18.25 – 33.68	21.32 – 45.83	11.94 – 17.52	18.71 – 35.4	8.68 – 21.92
**AQP1 - medulla**						
Mean ± SD	12.91 ± 4.54	11.52 ± 5.36	15.31 ± 8.38	13.7 ± 6.8	11.46 ± 5.19	12.61 ± 4.77
Median	12.53	11.39	14.1	12.04	10.09	12.37
Q1-Q3	9.13 – 15.03	7.77 – 14.81	9.44 – 21.54	8.12 – 18.65	8.23 – 12.43	8.36 – 15.57
**AQP2 - medulla**						
Mean ± SD	4.37 ± 1.39	3.62 ± 2.26	1.77 ± 1.58	1.054 ± 0.99	1.18 ± 0.58	2.07 ± 1.81
Median	4.16	3.52	1.32$^{*\#}$	0.69*	1.1	2.12$^{\Delta}$
Q1-Q3	3.45 – 5.06	1.61 – 5.69	0.71 – 1.99	0.21 – 1.77	0.82 – 1.51	0.35 – 3.38
**AVPR 2 - medulla**						
Mean ± SD	2.16 ± 0.83	1.08 ± 0.44	0.91 ± 0.43	0.96 ± 0.23	0.89 ± 0.46	1.22 ± 1.83
Median	1.95	1.11*	0.97*	0.92*	0.73	0.37
Q1-Q3	1.42 – 2.76	0.73 – 1.41	0.58 – 1.27	0.82 – 1.1	0.55 – 1.39	0.19 – 1.19
**SLC22A7 - cortex**						
Mean ± SD	13.45 ± 5.75	4.7 ± 3.2*	5.29 ± 1.71*	7.54 ± 3.05*	3.17 ± 1.8$^{\Delta}$	10.99 ± 3.39$^{\Delta\&}$
Median	12.94	3.87	5.16	6.94	2.67	11.87
Q1-Q3	11.03 – 16.03	2.15 – 6.87	4.57 – 5.89	5.29 – 9.02	1.69 – 4.42	8.39 – 13.65
**SLC22A7 - medulla**						
Mean ± SD	43.82 ± 4.27	16.61 ± 6.29	21.47 ± 9.43	28.2 ± 10.98	39.06 ± 6.77	25.75 ± 10.66
Median	43.13	17.65*	21.46*	26.17*	39.09$^{\Delta}$	25.02$^{\&}$
Q1-Q3	41.06 – 46.87	11.11 – 21.37	11.53 – 30.7	19.28 – 36.31	36.04 – 42.09	18.99 – 33.36
**Acetyl CoA cortex**						
Mean ± SD	5.15 ± 4.96	7.24 ± 5.65	3.36 ± 1.62	2.36 ± 1.03	2.97 ± 1.17	5.61 ± 1.73
Median	3.14	5.94*	3.1$^{\#}$	2.24*	2.81	5.45$^{\Delta\&}$
Q1-Q3	1.57 – 6.89	3.4 – 8.84	2.14 – 4.57	1.52 – 3.17	1.97 – 3.39	4.23 – 6.29
**Acetyl CoA medulla**						
Mean ± SD	16.41 ± 3.02	14.1 ± 3.76	5.78 ± 3.56	7.52 ± 2.93	12.91 ± 2.11	26.2 ± 5.5
Median	16.74	13.25	5.6$^{*\#}$	7.11*	12.99$^{\Delta}$	26.8$^{\Delta\&}$
Q1-Q3	14.41 – 17.89	11.17 – 17.18	2.94 – 8.25	5.78 – 8.95	12.13 – 14.6	23.39 – 29.02
**SREBP-1 cortex**						
Mean ± SD	4.98 ± 4.2	1.48 ± 1.33	4.62 ± 1.34	5.29 ± 1.99	1.9 ± 0.94	2.7 ± 1.16
Median	4.3	1.12*	4.77$^{\#}$	4.76*	1.7$^{\Delta}$	2.45$^{\Delta}$
Q1-Q3	0.96 – 8.04	0.55 – 1.95	3.61 – 5.58	3.7 – 7.32	1.26 – 2.38	1.74 – 3.38

**SREBP-1 medulla**						
Mean ± SD	8.77 ± 4.46	4.63 ± 1.53	2.75 ± 2.35	12.01 ± 2,46	2.99 ± 1.61	5.17 ± 2.27
Median	8.41	4.58*	1.66*#	12.11*	2.68Δ	4.62Δ&
Q1-Q3	6.07 – 10.86	3.78 – 5.28	1.28 – 3.21	10.38 – 12.84	1.98 – 3.6	3.5 – 6.61

Data are expressed as mean ± SD (standard deviation), M (median), Q1-Q2 (lower and upper quartile) of 6 rats in each group: c-C (control); C-IS (control supplemented with soy isoflavones); C-IS+IN (control supplemented with soy isoflavones plus inulin); c-DM (with induced diabetes mellitus; DM-IS (with induced diabetes mellitus supplemented with soy isoflavones); DM-IS+IN (with induced diabetes mellitus supplemented with soy isoflavones plus inulin). *$p < 0.05$ vs. c-C, Δ$p < 0.05$ vs. c-DM, #$p < 0.05$ vs. C-IS, &$p < 0.05$ vs. DM-IS. Significances associated with Mean±SD - parametric Fisher test (Tukey's post-hoc test); Significances associated with Median - non-parametric Kruskal-Wallis test (Dunn's post-hoc test).

Table S4. Kidney fatty acid profiles in each subgroup of rats.

	Control group			Diabetes group		
**Fatty acids (%)**	**c-C**	**C-IS**	**C-IS+IN**	**c-DM**	**DM-IS**	**DM-IS+IN**
**C14:0 Myristic**						
Mean ± SD	0.053±0.017	0.057±0.008	0.09±0.035	0.118±0.09	0.129±0.068	0.066±0.015
Median	0.047	0.06	0.08	0.076[c]	0.13	0.066
Q1-Q3	0.043–0.053	0.051-0.062	0.065–0.105	0.062–0.14	0.059–0.162	0.051–0.079
**C15:0 Pentadecanoid**						
X ± SD	0.038±0.008	0.037±0.006	0.056±0.021	0.038±0.015	0.041±0.024	0.028±0.012
Median	0.037	0.036	0.056	0.035	0.031	0.027
Q1-Q3	0.036–0.039	0.034–0.041	0.037–0.063	0.029–0.039	0.027–0.055	0.017–0.039
**C16:0 Palmitic**						
X ± SD	2.523±0.19	2.574±0.121	3.28±0.841	3.979±2.556	3.932±1.326	2.721±0.448
Median	2.482	2.629	3.003	2.944	4.032	2.688
Q1-Q3	2.427–2.554	2.458–2.662	2.618–3.674	2.732–3.415	2.687–4.845	2.335–2.797
**C16:1 Palmitoleic**						
X ± SD	0.047±0.016	0.044±0.007	0.074±0.016	0.146±0.175	0.12±0.11	0.041±0.011
Median	0.042	0.044	0.07	0.059	0.068	0.039
Q1-Q3	0.039–0.045	0.036–0.049	0.06–0.091	0.052–0.182	0.035–0.211	0.033–0.052
**C17:0 Heptadecanoic**						
X ± SD	0.075±0.013	0.066±0.006	0.01±0.068	0.06± 0.038	0.073±0.019	0.047±0.008
Median	0.069	0.067	0.075	0.058	0.078	0.049[Δ]
Q1-Q3	0.068–0.09	0.06–0.071	0.063–0.097	0.054–0.076	0.065–0.088	0.04–0.054
**C18:0 Stearic**						
X ± SD	2.67±0.069	2.746±0.154	2.835±0.284	3.136±0.725	3.282±0.46	2.9±0.07
Median	2.69	2.764	2.714	2.858	3.2	2.891
Q1-Q3	2.672–2.712	2.604–2.895	2.682–2.935	2.816–3.041	2.906 –3.635	2.844–2.936
**C18:1n9 Oleic**						
X ± SD	1.157±0.238	1.082±0.153	2.196±1.194	1.742±0.365	3.8±2.866[b]	1.405±0.237[&]
Median	1.052	1.053	2.016	1.659	2.682	1.394
Q1-Q3	1.027–1.31	0.967–1.139	1.207–2.848	1.44–2.034	1.622–6.5	1.186–1.561
**C18:1 tans vaccinic**						
X ± SD	0.343±0.031	0.334±0.011	0.449±0.131	0.544±0.432	0.516±0.216	0.034±0.04
Median	0.333	0.331	0.423	0.368	0.462	0.333
Q1-Q3	0.324–0.346	0.33–0.342	0.342–0.508	0.367–0.393	0.315–0.703	0.319–0.362
**C18:2n6c Linoleic**						
X ± SD	1.865±0.241	1.897±0.136	2.742±1.138	2.447±1.74	2.574±0.863	1.766±0.154
Median	1.817	1.92	2.529	1.74	2.375	1.799
Q1-Q3	1.738–1.857	1.888–1.977	1.893–3.43	1.604–2.064	1.944–3.262	1.604–1.871
**C18:3n6 γ linoleic**						
X ± SD	0.022±0.011	0.021±0.003	0.031±0.008	0.029±0.011	0.03±0.016	0.022±0.011
Median	2.69	0.021	0.029	0.028	0.0247	0.02
Q1-Q3	0.013–0.025	0.019–0.0222	0.025–0.04	0.019–0.04	0.017–0.041	0.013–0.025

**C18:3n3 linolenic**						
X ± SD	0.02±0.006	0.036±0.004	0.116±0.078	0.074±0.098	0.078±0.058	0.02±0.006
Median	0.002	0.036	0.102	0.03	0.067	0.0023[&]
Q1-Q3	0.013–0.024	0.035–0.038	0.048–0.166	0.024–0.065	0.027–0.126	0.013–0.024
**C20:3n6 eicosatrienoic**						
X ± SD	0.114±0.018	0.131±0.025	0.117±0.02	0.125±0.018	0.121±0.029	0.114±0.018
Median	0.109	0.128	0.119	0.13	0.118	0.109
Q1-Q3	0.101–0.116	0.116–0.14	0.097–0.136	0.116–0.132	0.106–0.131	0.101–0.116
**C20:4n6 Arachidonic**						
X ± SD	0.428±0.204	0.377±0.058	0.402±0.072	0.53±0.403	0.546±0.276	0.428±0.204
Median	0.376	0.373	0.399	0.406	0.421	0.376
Q1-Q3	0.305–0.415	0.323–0.438	0.362–0.458	0.356–0.415	0.351–0.807	0.305–0.415
**C20:5n3 EPA**						
X ± SD	0.021±0.007	0.029±0.013	0.039±0.011	0.045±0.039	0.039±0.276	0.021±0.007
Median	0.02	0.026	0.042	0.032	0.034	0.02
Q1-Q3	0.015–0.023	0.022–0.043	0.028–0.045	0.03–0.04	0.021–0.047	0.015–0.023
**C22:6n3 DHA**						
X ± SD	0.233±0.059	0.258±0.021	0.268±0.046	0.264±0.02	0.246±0.061	0.233±0.059
Median	0.227	0.252	0.267	0.263	0.23	0.227
Q1-Q3	0.173–0.295	0.24–0.282	0.255–0.282	0.251–0.276	0.196–0.316	0.173–0.295
**Parameter**						
**∑-SFA**						
X ± SD	5.359±0.247	5.479±0.247	6.362±1.237	7.332±3.394	7.457±1.84	5.761±0.513
Median	5.306	5.525	5.916	6.005	7.469	5.711
Q1-Q3	5.156–5.438	5.335–5.648	5.455–6.857	5.772–6.32	5.898–8.658	5.339–5.884
**∑-PUFA**						
X ± SD	2.676±0.392	2.75±0.159	3.715±1.306	3.513±1.79	3.634±0.897	2.603±0.345
Median	2.6	2.74	3.44	2.748	3.744	2.536
Q1-Q3	2.421–2.674	2.653–2.867	2.648–4.539	2.466–3.597	3.034–4.016	2.426–2.725
**∑-MUFA**						
X ± SD	1.548±0.279	1.46±0.158	2.719±1.332	2.433±0.628	4.435±3.178	1.788±0.28[&]
Median	1.42	1.424	2.517	2.307	3.212	1.791
Q1-Q3	2.404–1.692	1.357–1.522	1.596–3.447	1.905–2.737	1.97–7.501	1.531–1.925
**∑-UFA**						
X ± SD	4.224±0.656	4.21±0.226	6.434±2.637	5.945±2.384	8.07±3.991	4.391±0.566[&]
Median	4.049	4.279	5.962	5.056	6.861	4.326
Q1-Q3	3.735–4.308	3.945–4.334	4.244–7.986	4.371–6.335	5.194–11.517	3.957–4.954
**PUFA/SFA**						
X ± SD	0.498±0.051	0.502±0.011	0.571±0.104	0.474±0.053	0.489±0.054	0.478±0.06
Median	0.486	0.502	0.582	0.458	0.47	0.445
Q1-Q3	0.469–0.508	0.496–0.508	0.485–0.664	0.434–0.496	0.464–0.494	0.434–0.555
**∑-n-6**						
X ± SD	2.365±0.353	2.427±0.146	3.292±1.219	3.131±1.692	3.271±0.865	2.33±0.344
Median	2.285	2.426	3.08	2.424	3.354	2.233
Q1-Q3	2.124–2.37	2.352–2.524	2.319–4.046	2.16–3.17	2.635–3.69	2.16–2.378

**∑-n-3**						
X ± SD	0.293±0.03	0.308±0.019	0.338±0.052	0.338±0.038	0.315±0.072	0.275±0.055
Median	0.3	0.302	0.347	0.328	0.317	0.271
Q1-Q3	0.278–0.31	0.294–0.33	0.302–0.366	0.319–0.353	0.266–0.383	0.237–0.333
**n-6/n-3**						
X ± SD	8.077±0.754	7.883±0.368	9.779±3.147	9.32±5.142	11.053±4.719	8.749±2.205
Median	7.771	7.987	10.199	7.511	9.031	8.084
Q1-Q3	7.643–8.58	7.65–8.157	7.685–11.414	7.34–7.848	7.483–17.025	7.033–9.855
**SCD16**						
X ± SD	0.018±0.004	0.017±0.003	0.023±0.005	0.032±0.022	0.027±0.02	0.015±0.003
Median	0.017	0.018	0.024	0.019	0.017	0.015
Q1-Q3	0.017–0.019	0.014–0.019	0.021–0.026	0.018–0.053	0.015–0.037	0.013–0.016
**SCD18**						
X ± SD	0.434±0.091	0.396±0.068	0.752±0.34	0.579±0.181	1.079±0.685	0.484±0.075$^{\&}$
Median	0.392	0.389	0.751	0.561	0.823	0.485
Q1-Q3	0.379–0.517	0.341–0.406	0.45–0.97	0.47–0.669	0.558–1.788	0.408–0.542
**D6D**						
X ± SD	0.014±0.007	0.011±0.002	0.012±0.004	0.014±0.007	0.013±0.009	0.012±0.006
Median	0.011	0.011	0.012	0.014	0.01	0.011
Q1-Q3	0.009–0.002	0.01–0.012	0.009–0.016	0.008–0.019	0.007–0.016	0.008–0.015
**DNL**						
X ± SD	1.361±0.083	1.36±0.06	1.277±0.275	1.676±0.224^{c}	1.528±0.172	1.542±0.213
Median	1.357	1.342	1.226	1.608	1.467	1.51
Q1-Q3	1.334–1.394	1.316–1.403	1.063–1.383	1.534–1.754	1.449–1.539	1.379–1.665
**Elovl-5**						
X ± SD	5.667±2.585	6.407±1.661	3.936±0.873	5.007±2.224	4.569±1.391	6.045±2.24
Median	5.766	5.734	3.637	4.461	4.703	5.778
Q1-Q3	3.684–8.026	5.235–7.521	3.258–4.859	3.525–6.733	3.0–5.671	4.052–8.376
**Elovl-6**						
X ± SD	1.062±0.072	1.067±0.493	0.891±0.128	0.897±0.22	0.89±0.204	1.084±0.146
Median	1.067	1.074	0.91	0.997	0.838	1.059
Q1-Q3	1.032–1.1	1.057–1.108	0.799–0.992	0.777–1.055	0.704–1.081	1.05–1.243
**Elongation FA**						
X ± SD	1.518±0.04	1.487±0.024	1.525±0.098	1.424±0.374	1.757±0.314	1.601±0.148
Median	1.511	1.486	1.545	1.574	1.698	1.617
Q1-Q3	1.49–1.563	1.485–1.492	1.488–1.574	1.452–1.596	1.458–2.131	1.532–1.737

Data are expressed as mean ± SD (standard deviation), M (median), Q1-Q3 (lower and upper quartile of 6 rats in each group: c-C (control); C-IS (control supplemented with soy isoflavones); C-IS+IN (control supplemented with soy isoflavones plus inulin); c-DM (with induced diabetes mellitus; DM-IS (with induced diabetes mellitus supplemented with soy isoflavones); DM-IS+IN (with induced diabetes mellitus supplemented with soy isoflavones plus inulin). $^{\Delta}p < 0.05$ vs. c-DM, $^{\&}p < 0.05$ vs. DM-IS, $^{b}p < 0.055$ vs. c-DM, and $^{c}p < 0.055$ vs. c-C – on the border of statistically significant differences. Significances associated with Mean±SD - parametric Fisher test (Tukey's post-hoc test); Significances associated with Median - non-parametric Kruskal-Wallis test (Dunn's post-hoc test).
